# Associations of maternal bisphenol urine concentrations during pregnancy with neonatal metabolomic profiles

**DOI:** 10.1007/s11306-021-01836-w

**Published:** 2021-09-13

**Authors:** Sophia M. Blaauwendraad, Ellis Voerman, Leonardo Trasande, Kurunthachalam Kannan, Susana Santos, George J. G. Ruijter, Chalana M. Sol, Linda Marchioro, Engy Shokry, Berthold Koletzko, Vincent W. V. Jaddoe, Romy Gaillard

**Affiliations:** 1 The Generation R Study Group (Na-29), Erasmus MC, University Medical Center, PO Box 2040, 3000 CA Rotterdam, the Netherlands; 2 Department of Pediatrics, Erasmus MC, University Medical Center, Rotterdam, the Netherlands; 3 Department of Paediatrics, New York University School of Medicine, New York City, NY 10016 USA; 4 Department of Environmental Medicine, New York University School of Medicine, New York City, NY 10016 USA; 5 Department of Population Health, New York University School of Medicine, New York City, NY USA; 6 School of Public Service, New York University Wagner, New York City, NY 10016 USA; 7 New York University College of Global Public Health, New York City, NY 10016 USA; 8 Department of Clinical Genetics, Center for Lysosomal and Metabolic Disease, Erasmus MC, University Medical Center, Rotterdam, the Netherlands; 9 Division of Metabolic and Nutritional Medicine, Dr. Von Hauner Children’s Hospital, LMU—Ludwig-Maximilians Universität München, Munich, Germany

**Keywords:** Bisphenol, Metabolomics, Phospholipids, Carnitines, Fatty acids, Pregnancy

## Abstract

**Background:**

Fetal exposure to bisphenols is associated with altered fetal growth, adverse birth outcomes and childhood cardio-metabolic risk factors. Metabolomics may serve as a tool to identify the mechanisms underlying these associations. We examined the associations of maternal bisphenol urinary concentrations in pregnancy with neonatal metabolite profiles from cord blood.

**Methods:**

In a population-based prospective cohort study among 225 mother–child pairs, maternal urinary bisphenol A, S and F concentrations in first, second and third trimester were measured. LC–MS/MS was used to determine neonatal concentrations of amino acids, non-esterified fatty acids (NEFA), phospholipids (PL), and carnitines in cord blood.

**Results:**

No associations of maternal total bisphenol concentrations with neonatal metabolite profiles were present. Higher maternal average BPA concentrations were associated with higher neonatal mono-unsaturated alkyl-lysophosphatidylcholine concentrations, whereas higher maternal average BPS was associated with lower neonatal overall and saturated alkyl-lysophosphatidylcholine (p-values < 0.05).Trimester-specific analyses showed that higher maternal BPA, BPS and BPF were associated with alterations in neonatal NEFA, diacyl-phosphatidylcholines, acyl-alkyl-phosphatidylcholines, alkyl-lysophosphatidylcholine, sphingomyelines and acyl-carnitines, with the strongest effects for third trimester maternal bisphenol and neonatal diacyl-phosphatidylcholine, sphingomyeline and acyl-carnitine metabolites (p-values < 0.05). Associations were not explained by maternal socio-demographic and lifestyle characteristics or birth characteristics.

**Discussion:**

Higher maternal bisphenol A, F and S concentrations in pregnancy are associated with alterations in neonatal metabolite profile, mainly in NEFA, PL and carnitines concentrations. These findings provide novel insight into potential mechanisms underlying associations of maternal bisphenol exposure during pregnancy with adverse offspring outcomes but need to be replicated among larger, diverse populations.

**Supplementary Information:**

The online version contains supplementary material available at 10.1007/s11306-021-01836-w.

## Introduction

The plastic monomers and plasticizers bisphenol A (BPA), bisphenol F (BPF) and bisphenol S (BPS) are among the most produced chemical compounds worldwide and are widely used in the production of common consumer goods such as plastic bottles, food can coatings and thermal paper products (Hormann et al., 2014; Liao & Kannan, 2014; Liao et al., 2012; Vandenberg et al., 2010). Similar to the general population, pregnant women are regularly exposed to bisphenols (Woodruff et al., 2011; Ye et al., 2008). Accumulating evidence suggests that maternal exposure in pregnancy to these endocrine-disrupting chemicals, that can freely cross the placenta, may influence fetal growth, cardio-metabolic development and metabolism (Goldinger et al., 2015; Nahar et al., 2015; Stillerman et al., 2008). Observational studies have shown that higher maternal exposure to BPA, BPF and BPS are associated with altered fetal growth patterns and increased risks of both low and high birth weight (Ferguson et al., 2018; Hu et al., 2018, 2019; Zhong et al., 2020; Zhou et al., 2019). Higher maternal exposure to BPA and to a lesser extent BPF and BPS may also be associated with a higher childhood body mass index (BMI), waist circumference, blood pressure, and risk of overweight, although findings across studies are inconsistent (Harley et al., 2013; Lee et al., 2008, 2014; Philippat et al., 2014; Sol et al., 2020; Valvi et al., 2013).

The mechanisms underlying these associations of bisphenol exposure with adverse birth outcomes and adverse cardio-metabolic profiles in later life are not well-known but may involve alterations in metabolism. Higher fetal and childhood bisphenol exposure are associated with increased plasma levels of conventional metabolic biomarkers such as leptin, cholesterol and insulin, and with higher insulin resistance (Carlsson et al., 2018; Khalil et al., 2014; Volberg et al., 2013). With metabolomics techniques, a detailed characterization of fetal metabolic profiles can be obtained, enabling more in-depth insight into potential underlying metabolic mechanisms (Tzoulaki et al., 2014). Among adult populations, it has already been shown that higher BPA exposure is associated with changes in the amino-acid (AA) metabolism, fatty acids (FA) elongation and sphingolipid metabolism (Cho et al., 2018; Khan et al., 2017). Also, animal studies have demonstrated that fetal exposure to high BPA levels is associated with alterations in neonatal urinary and serum metabolome, characterized by changes in various AA, low-density lipoproteins, very low-density lipoproteins, choline, glucose and glycogen levels, but no studies on the association of fetal exposure to bisphenols with neonatal metabolite profiles among human populations have been performed (Cabaton et al., 2013; Meng et al., 2019a, 2019b, 2019c; Tremblay-Franco et al., 2015).

Therefore, in a subgroup of a population-based prospective cohort from early pregnancy onwards among 225 mothers-child pairs, we assessed the associations of maternal bisphenol A, S and F urinary concentrations throughout pregnancy with neonatal metabolite profiles obtained from cord blood.

## Methods

### Study design and population

**Fig. 1:**
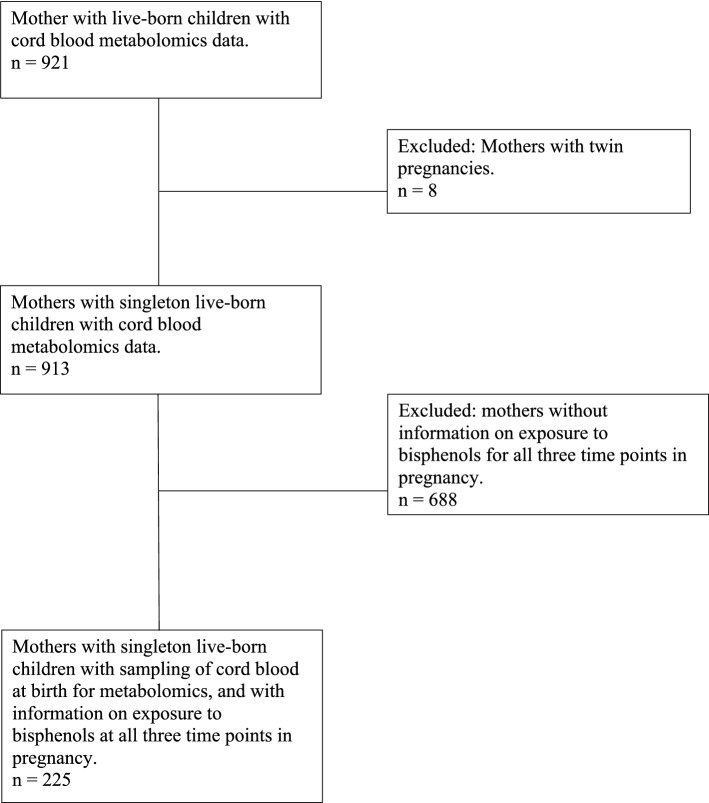
Flowchart of participants included in the study

This study is embedded in the Generation R Study, a population-based prospective cohort study from fetal life until adulthood in Rotterdam, the Netherlands (Kooijman et al., 2016). Study approval was obtained by the Medical Ethical Committee of the Erasmus Medical Center, University Medical Center, Rotterdam (MEC 198.782/2001/31). Written informed consent was obtained from all mothers. For the metabolomics analyses, cord blood metabolomic data were available for a subsample of 921 live-born children, of whom 913 were singleton. Of these, 225 mothers had bisphenol urine concentration measurements available at three time points in pregnancy (Fig. 1).

### Maternal bisphenol measurement

Maternal bisphenol concentrations (Bisphenol A (BPA), S (BPS), F (BPF), Z (BPZ), B (BPB), AP (BPAP), P (BPP) and AF (BPAF)) were measured in spot urine samples obtained from each woman at three time points during pregnancy [median 12.6 weeks of gestation (95% range 9.8–16.8); median 20.4 weeks of gestation (95% range 19.0–22.8); median 30.2 weeks of gestation (95% range 28.2–32.5)]. The bisphenol and creatinine analyses were performed at the Wadsworth Center, New York State Department of Health, Albany, New York, USA. Details on collection, transportation and analysis methodology are provided elsewhere (Philips et al., 2018). Individual bisphenols were assessed individually and grouped as a proxy for total bisphenol exposure when ≥ 20% of the samples was above the limit of detection (LOD) (LOD per bisphenol shown in Supplementary Table S1). The LOD was calculated as 3S_0_, where S_0_ is the standard deviation as the concentration approaches zero (Calafat et al., 2008). The LOD is the concentration at which a measurement has a 95% probability of being greater than zero. We selected the LOD cut-off of 20% because with this cut-off we were able to include the maximum number of participants in the analyses with adequate variability in the bisphenol data to detect associations. This approach is in line with previous studies in the field (Philips et al., 2018; Sol et al., 2020; van den Dries et al., 2020). Bisphenols A, S and F met those inclusion criteria (Supplementary Table S1). Concentrations below LOD were imputed by the LOD of that compound divided by the square root of 2 (LOD/√2) (Hornung & Reed, 1990). To account for urinary dilutions, molar sums or weighted molar sums in μmol/g creatinine were calculated for the individual and grouped bisphenols respectively. The bisphenol and creatinine analyses were performed at the Wadsworth Center, New York State Department of Health, Albany, New York, USA (Philips et al., 2018). The descriptive statistics of the individual and grouped bisphenols investigated are shown in Supplementary Table S2. Within individual variability of the bisphenols was assessed in a previous study, concluding low intraclass correlations (Sol et al., 2020). To reduce the potential for exposure misclassification due to temporal variability, we calculated the overall mean exposure during pregnancy. We also explored trimester specific effects.

For all analyses, urine bisphenol concentrations were natural log-transformed to reduce variability and account for right skewedness of the distribution and further standardized by the interquartile range (IQR) to ease interpretation of the effect estimates.

### Metabolite measurement

As described in detail previously, umbilical venous cord blood samples for metabolomics analyses were collected directly after birth [median gestation age at birth 40.4 weeks (95% range 37.3–42.3)] by a midwife or obstetrician (Voerman et al., 2020). Blood samples were transported to the regional laboratory (STAR-MDC), spun and stored at − 80 °C within 4 h after collection. They were transported on dry ice to the Division of Metabolic and Nutritional Medicine of the Dr. von Hauner Children’s Hospital in Munich, Germany.

As described in detail previously (Hellmuth et al., 2017; Voerman et al., 2020), a targeted metabolomics approach was used to determine the serum concentrations (µmol/L) of AA, non-esterified fatty acids (NEFA), phospholipids (PL) [including diacyl-phosphatidylcholines (PC.aa), acyl-alkyl-phosphatidylcholines (PC.ae), acyl-lysophosphatidylcholines (Lyso.PC.a), alkyl-lysophosphatidylcholines (Lyso.PC.e), sphingomyelines (SM)] and carnitines (Carn) [including free carnitine (Free Carn) and acyl-carnitines (Carn.a)]. Proteins of 50 µL serum were precipitated by adding 450 µL methanol with the following internal standards: labeled amino acid standards set A (NSK-A-1, Cambridge Isotope Laboratories (CIL), USA), 15N2-L-asparagine (NLM-3286-0.25, CIL, USA), indole-D5-L-tryptophan (DLM-1092-0.5, CIL, USA), U-13C16-palmitic acid (CLM-409-MPT-PK, CIL, USA), D3-acetyl-carnitine (DLM-754-PK, CIL, USA), D3-octanoyl-carnitine (DLM-755-0.01, CIL, USA), and D3-palmitoyl-carnitine (DLM-1263-0.01, CIL, USA), tridecanoyl-2-hydroxy-sn-glycero-3-phosphocholine (855476, Avanti Polar Lipids, USA) and 1,2-dimyristoyl-sn-glycero-3-phospocholine (850345, Avanti Polar Lipids, USA) (Voerman et al., 2020). If sample volume was less than optimal, the concentrations were corrected by the respective factor. Sample volumes less than 25 µL were considered missing. After centrifugation, we split the supernatant into aliquots. We analyzed AA by liquid chromatography tandem mass spectrometry (LC–MS/MS), as described previously (Harder et al., 2011). An aliquot of the supernatant was used for the derivatization to AA butylester with hydrocholic acid in 1-buthanol. After evaporation, the residues were dissolved in water/methanol (80:20; (v/v)) with 0.1% formic acid (Voerman et al., 2020). The samples were analyzed with 1100 high-performance liquid chromatography (HPLC) system (Agilent, Waldbronn, Germany) equipped with 150 × 2.1 mm, 3.5 µm particle size C18 HPLC column (X-Bridge, Waters, Milford, USA) and 0.1% heptafluorobutyric acid as an ion pair reagent in the mobile phases A (water) and B (methanol). We performed mass spectrometry (MS) detection with an API2000 tandem mass spectrometer (MS/MS) (AB Sciex, Darmstadt, Germany). IUPAC-IUB Nomenclature was used for notation of AA [(JCBN) 1984]. For AA, information on the identification and analysis for each metabolite and class are presented in Supplementary Table S3.

NEFA, PL and Carn were measured with a 1200 SL HPLC system (Agilent, Waldbronn, Germany) coupled to a 4000 QTRAP tandem mass spectrometer (AB Sciex, Darmstadt, Germany) (Hellmuth et al., 2012; Uhl et al., 2016). NEFA were analyzed by injection of the supernatant to a LC–MS/MS operating in negative electrospray ionization (ESI) mode where they separated by gradient elution on a 100 × 3.0 mm, 1.9 µm particle size Purusuit UPS Diphenyl column from Varian (Darmstadt, Germany) using 5 mM ammonium acetate in water as mobile phase A and acetonitrile/isopropanol [80:20, (v/v)] as mobile phase B (Voerman et al., 2020). NEFA species were quantified using GLC-85 reference standard mixture (Nu-Chek Prep, USA). For NEFA, information on the identification and analysis for each metabolite and class are presented in Supplementary Table S3.

PL were analyzed by flow-injection-analysis with LC–MS/MS coupled with ESI (Rauschert et al., 2016). The system was run in positive ionization mode with 5% water in isopropanol as mobile phase A and 5% water in methanol as mobile phase B. The method included 2 periods of 2.6 min each. The total runtime for both periods was 5.2 min and 0.8 injection time with a total injection volume of 60 µL. The analysis was performed for PC.aa, PC.ae, Lyso.PC.a, Lyso.PC.e and SM. For Carn (Free Carn and Carn.a) analysis we performed flow-injection analysis of the supernatant into a LC–MS/MS system using an isocratic elution with 76% isopropanol, 19% methanol and 5% water (Voerman et al., 2020). The mass spectrometer was equipped with electrospray ionization and operated in the positive ionization mode. PL and Carn.a were quantified using aliquots of a commercially available lyophilized control plasma (ClinChek®, Recipe, Germany), where the concentrations have been determined by AbsoluteIDQ p150 Kit from Biocrates®, a previous published LC–MS/MS method and by in-house quantification with various standards (Uhl et al., 2011). Information on the identification and analysis of PL and Carn.a are given in Supplementary Table S4 (Voerman et al., 2020). The entire analytical process was controlled and post-processed by Analyst 1.6.1. and R Software (Hellmuth et al., 2017). The analytical technique used can determine the total number of total bonds, but not the position of the double bonds and the distribution of the carbon atoms between FA side chains. The following notation was used for NEFA, PL and Carn.a: X:Y, where X denotes the length of the carbon chain, and Y the number of double bonds. The ‘a’ denotes an acyl chain bound to the backbone of an ester bond (‘acyl-’) and the ‘e’ represents an ether bond (‘alkyl-’).

Data quality control (QC) was based on thresholds of 25% and 35% for the intra- and inter-batch coefficients of variation respectively (Voerman et al., 2020). To correct for batch effects, metabolite concentrations were divided by the ratio of the intra-batch and inter-batch median of the QC samples. Metabolites and participants with more than 50% of missing values were excluded. Missing metabolite values of the remaining metabolites and participants were imputed using the Random Forest algorithm (R package *missForest*), which is among the best performing imputation methods for mass-spectrometry based metabolomics data with missing values at random or missing values completely at random (Hellmuth et al., 2017; Shokry et al., 2019; Wei et al., 2018). The Random Forest algorithm works by aggregating the predictions made by multiple decision trees of varying depth. The trees (or models) are relatively uncorrelated, as each tree samples at random from the dataset and the trees use different features to make the decision instead of always picking the feature that provides the most separation.

For analyses, we categorized metabolites into general metabolite groups based on chemical structure (AA, NEFA, PC.aa, PC.ae, Lyso.PC.a, Lyso.PC.e, SM, Free Carn and Carn.a) and in detailed metabolite subgroups based on chemical structure and biological relevance (AA: branched chain AA (BCAA), aromatic AA (AAA), essential AA, non-essential AA; NEFA, PC.aa, PC.ae, Lyso.PC.a, Lyso.PC.e and SM: saturated, mono-unsaturated, poly-unsaturated; Carn.a: short-chain, medium-chain, long-chain) (Voerman et al., 2020). Correlations between metabolites were assessed in a previous study, concluding high correlations between individual metabolites within groups of metabolites with similar chemical structures, but lower correlations between groups of metabolites with different chemical structures (Voerman et al., 2020). To correct for right skewedness, individual metabolite concentrations were square root transformed. To facilitate interpretation of the effect estimates, standard deviation scores (SDS) were calculated for both metabolite groups and individual metabolites.

### Covariates

Information on maternal age, ethnicity, pre-pregnancy BMI, educational level, total energy intake and parity was obtained at enrollment through questionnaires (Kooijman et al., 2016). We assessed maternal smoking and alcohol consumption during pregnancy through questionnaires in each trimester. Information on the child’s sex, gestational age at birth and birthweight was obtained from medical records.

### Statistical analysis

First, we performed two non-response analysis comparing characteristics of mothers–child pairs with information on bisphenol concentrations in pregnancy and neonatal metabolomics to mother–child pairs without this information, respectively. Second, we examined the associations of average (e.g. summed concentrations of three trimesters divided by three) and trimester-specific maternal total bisphenol concentrations and bisphenol A, S and F concentrations with neonatal general metabolite groups and neonatal metabolite subgroups using linear regression models. These models were adjusted for maternal age, educational level, pre-pregnancy BMI, parity, smoking, alcohol use and total energy intake. These possible confounders were selected based on Directed Acyclic Graph (DAG) analysis and association with exposure and outcomes in existing literature (DAG shown in Supplementary Fig. 1) (Arbuckle et al., 2015; Casas et al., 2013; Philips et al., 2018; Ruoppolo et al., 2015; Syggelou et al., 2012; Taylor et al., 2019). All statistical tests were 2-sided. P-values for all analysis are presented. Nominal (p-value < 0.05), FDR-adjusted (p-value < 0.006 based on 8 metabolite groups) and Bonferroni-adjusted (p-value < 5.21 × 10^–5^ based on 960 linear regressions statistical significance thresholds were considered. For nominal significant associations with neonatal metabolite groups, we performed additional analyses: (1) we further explored the associations of maternal bisphenols with individual neonatal metabolites in the specific neonatal metabolite group; (2) we explored whether additional adjustment for fetal sex, birth weight and gestational age at birth explained the observed associations, as neonatal metabolic profiles correlate with these birth characteristics (Syggelou et al., 2012). Missing values of covariates were imputed using multiple imputation using 5 datasets. The analyses were performed using Statistical Package of Social Science version 25.0 (SPSS Inc., Chicago, IL, USA).

## Results

### Population characteristics

Table 1 shows the population characteristics. Summed cord blood metabolite groups and individual metabolite concentrations are shown in Supplementary Table S5. Non-response analysis showed that mothers without bisphenol measurements were more often multiparous and smoked more often in pregnancy, compared to mothers with bisphenol measurements (Supplementary Table S6). Their metabolite concentrations of AA, Lyso.PC.a, PC.aa, PC.ae, SM, free Carn and Carn.a tended to be higher (Supplementary Table S7). Children without cord blood sampling for metabolomics showed no important differences compared to children with cord blood sampling for metabolomics (Supplementary Table S8).

**Table 1:** General characteristics of study population

	Total sample n = 225
Maternal characteristics	
Age at enrolment, mean (± SD), years	31.9 (3.7)
Parity, *n* (%)	
Nullipara	150 (66.7)
Multipara	75 (33.3)
Ethnicity, *n* (%)	
Dutch	225 (100)
Other	0 (0)
Education, *n* (%)	
Primary	3 (1.3)
Secondary	76 (33.8)
Higher	145 (64.4)
Pre-pregnancy body mass index, median (95% range), kg/m^2^	22.6 (18.6–35.6)
Smoking, *n* (%)	
Never smoked during pregnancy	161 (71.6)
Smoked until pregnancy was known	14 (6.2)
Continued smoking in pregnancy	19 (8.4)
Alcohol consumption, *n* (%)	
Never alcohol in pregnancy	53 (23.6)
Alcohol until pregnancy was known	38 (16.9)
Alcohol continued in pregnancy	103 (45.8)
Child characteristics	
Gestational age at birth in weeks, median (95% range)	40.4 (37.3–42.3)
Premature birth, *n* (%)	3 (1.3)
Sex, *n* (%)	
Male	129 (57.3)
Female	96 (42.7)
Birthweight, mean (± SD), gram	3538.8 (453.5)
SGA, *n* (%)	22 (9.8)
LGA, *n* (%)	22 (9.8)
Low birthweight, *n* (%)	2 (0.9)
Macrosomia, *n* (%)	31 (13.8)

Values represent mean (± SD), median (95% range) or number of participants (valid %)

Premature birth, birth at gestational age < 37 weeks; SGA, small for gestational age, gestational age adjusted birthweight < 10th percentile; LGA, large for gestational age, gestational age adjusted birthweight > 90th percentil; low birthweight, birthweight < 2500 kg; macrosomia, birthweight > 4000 kg

### Maternal total bisphenol concentrations and neonatal metabolite profiles

No significant associations of higher maternal average or trimester-specific total bisphenol concentrations in urine with neonatal serum metabolite groups were present (Table 2).

**Table 2:** Associations of maternal average and trimester-specific total bisphenol exposure in pregnancy with cord blood metabolic groups and subgroups

Neonatal metabolite group	Maternal average total bisphenol		Maternal first trimester total bisphenol		Maternal second trimester total bisphenol		Maternal third trimester total bisphenol	
	Difference in neonatal metabolite group (SDS) (95% CI)	P-value	Difference in neonatal metabolite group (SDS) (95% CI)	P-value	Difference in neonatal metabolite group (SDS) (95% CI)	P-value	Difference in neonatal metabolite group (SDS) (95% CI)	P-value
Amino acids (AA)	− 0.05 (− 0.21, 0.12)	0.570	− 0.02 (− 0.18, 0.15)	0.856	− 0.11 (− 0.28, 0.06)	0.207	− 0.03 (− 0.19, 0.14)	0.753
Branched-chain AA	− 0.06 (− 0.22, 0.10)	0.436	− 0.06 (− 0.22, 0.10)	0.438	− 0.08 (− 0.25, 0.08)	0.324	− 0.02 (− 0.15, 0.18)	0.833
Aromatic AA	− 0.06 (− 0.22, 0.11)	0.508	− 0.03 (− 0.19, 0.13)	0.708	− 0.08 (− 0.25, 0.09)	0.334	− 0.02 (− 0.18, 0.14)	0.818
Essential AA	− 0.04 (− 0.20, 0.12)	0.640	− 0.06 (− 0.21, 0.11)	0.501	− 0.04 (− 0.20, 0.13)	0.681	− 0.01 (− 0.17, 0.15)	0.923
Non-essential AA	− 0.05 (− 0.21, 0.12)	0.565	0.01 (− 0.15, 0.17)	0.912	− 0.14 (− 0.31, 0.03)	0.106	− 0.04 (− 0.20, 0.13)	0.669
Non-esterified fatty acids (NEFA)	− 0.10 (− 0.26, 0.07)	0.245	− 0.05 (− 0.21, 0.11)	0.535	− 0.01 (− 0.17, 0.16)	0.948	0.00 (− 0.17, 0.16)	0.965
Saturated NEFA	− 0.10 (− 0.26, 0.06)	0.211	− 0.06 (− 0.22, 0.10)	0.474	0.00 (− 0.16, 0.17)	0.983	0.00 (− 0.16, 0.16)	0.986
Mono-unsaturated NEFA	− 0.08 (− 0.24, 0.08)	0.343	− 0.03 (− 0.19, 0.13)	0.707	− 0.02 (− 0.18, 0.15)	0.853	− 0.02 (− 0.18, 0.14)	0.829
Poly-unsaturated NEFA	− 0.09 (− 0.26, 0.07)	0.272	− 0.06 (− 0.22, 0.11)	0.499	0.00 (− 0.17, 0.17)	0.999	0.01 (− 0.15, 0.18)	0.866
Diacyl-phosphatidylcholines (PC.aa)	0.01 (− 0.15, 0.17)	0.920	− 0.04 (− 0.12, 0.20)	0.654	0.03 (− 0.14, 0.20)	0.725	− 0.07 (− 0.23, 0.09)	0.409
Saturated PC.aa	0.03 (− 0.13, 0.19)	0.714	0.02 (− 0.14, 0.18)	0.824	0.11 (− 0.06, 0.27)	0.209	− 0.04 (− 0.20, 0.12)	0.637
Mono-unsaturated PC.aa	0.05 (− 0.11, 0.21)	0.545	0.06 (− 0.10, 0.22)	0.466	− 0.11 (− 0.06, 0.28)	0.187	− 0.10 (− 0.26, 0.07)	0.245
Poly-unsaturated PC.aa	0.00 (− 0.16, 0.16)	0.985	0.03 (− 0.13, 0.19)	0.697	− 0.01 (− 0.16, 0.17)	0.929	− 0.06 (− 0.22, 0.10)	0.456
Acyl-alkyl-phosphatidylcholines (PC.ae)	− 0.03 (− 0.19, 0.14)	0.763	− 0.02 (− 0.17, 0.15)	0.859	0.01 (− 0.16, 0.18)	0.912	− 0.03 (− 0.19, 0.14)	0.753
Saturated PC.ae	− 0.06 (− 0.22, 0.10)	0.460	− 0.06 (− 0.22, 0.10)	0.445	0.07 (− 0.09, 0.24)	0.291	− 0.04 (− 0.20, 0.12)	0.610
Mono-unsaturated PC.ae	0.00 (− 0.16, 0.17)	0.967	− 0.02 (− 0.18, 0.14)	0.798	0.12 (− 0.04, 0.29)	0.144	− 0.07 (− 0.23, 0.09)	0.197
Poly-unsaturated PC.ae	− 0.02 (− 0.18, 0.14)	0.811	0.00 (− 0.16, 0.16)	0.980	− 0.02 (− 0.19, 0.14)	0.781	− 0.01 (− 0.18, 0.15)	0.873
Acyl-lysophosphatidylcholines (Lyso.PC.a)	0.04 (− 0.12, 0.20)	0.649	0.00 (− 0.16, 0.16)	0.991	0.03 (− 0.14, 0.19)	0.774	− 0.01 (− 0.17, 0.15)	0.888
Saturated Lyso.PC.a	0.06 (− 0.10, 0.23)	0.458	0.01 (− 0.15, 0.17)	0.901	− 0.01 (− 0.18, 0.16)	0.900	− 0.02 (− 0.14, 0.19)	0.784
Mono-unsaturated Lyso.PC.a	− 0.01 (− 0.18, 0.15)	0.868	− 0.02 (− 0.18, 0.14)	0.773	0.09 (− 0.08, 0.26)	0.303	− 0.11 (− 0.27, 0.05)	0.188
Poly-unsaturated Lyso.PC.a	0.00 (− 0.16, 0.16)	0.958	− 0.01 (− 0.16, 0.15)	0.955	0.08 (− 0.09, 0.24)	0.348	− 0.05 (− 0.21, 0.12)	0.582
Alkyl-lysophosphatidylcholines (Lyso.PC.e)	− 0.06 (− 0.22, 0.11)	0.504	− 0.03 (− 0.19, 0.14)	0.764	0.04 (− 0.12, 0.21)	0.612	− 0.07 (− 0.23, 0.09)	0.379
Saturated Lyso.PC.e	− 0.08 (− 0.24, 0.08)	0.333	− 0.04 (− 0.20, 0.12)	0.618	0.02 (− 0.15, 0.18)	0.852	− 0.09 (− 0.25, 0.07)	0.280
Mono-unsaturated Lyso.PC.e	0.09 (− 0.07, 0.25)	0.252	0.08 (− 0.08, 0.23)	0.353	0.16 (0.00, 0.32)	0.055	− 0.03 (− 0.13, 0.19)	0.757
Sphingomyelines (SM)	0.03 (− 0.13, 0.19)	0.714	0.02 (− 0.14, 0.18)	0.771	0.10 (− 0.06, 0.27)	0.224	− 0.03 (− 0.19, 0.13)	0.738
Mono-unsaturated SM	0.00 (− 0.16, 0.16)	0.995	− 0.03 (− 0.19, 0.13)	0.727	0.13 (− 0.04, 0.30)	0.125	− 0.03 (− 0.19, 0.13)	0.699
Poly-unsaturated SM	0.05 (− 0.11, 0.21)	0.509	0.07 (− 0.09, 0.23)	0.408	0.08 (− 0.09, 0.24)	0.367	− 0.02 (− 0.18, 0.14)	0.770
Free carnitine	0.05 (− 0.12, 0.21)	0.573	0.04 (− 0.12, 0.20)	0.635	0.00 (− 0.17, 0.17)	0.981	0.00 (− 0.17, 0.16)	0.979
Acyl-carnitines (Carn.a)	− 0.01 (− 0.17, 0.14)	0.860	0.00 (− 0.16, 0.15)	0.957	0.01 (− 0.16, 0.17)	0.952	0.01 (− 0.15, 0.17)	0.884
Small-chain Carn.a	− 0.03 (− 0.19, 0.13)	0.685	− 0.03 (− 0.18, 0.13)	0.750	0.00 (− 0.16, 0.17)	0.974	0.01 (− 0.15, 0.17)	0.889
Medium-chain Carn.a	0.08 (− 0.08, 0.23)	0.330	− 0.08 (− 0.07, 0.24)	0.293	0.05 (− 0.11, 0.21)	0.538	− 0.04 (− 0.20, 0.12)	0.646
Large-chain Carn.a	0.06 (− 0.10, 0.22)	0.447	− 0.09 (− 0.07, 0.25)	0.287	− 0.01 (− 0.17, 0.16)	0.952	− 0.04 (− 0.12, 0.21)	0.604

Values represent regression coefficients (95% confidence interval) and corresponding p-values from linear regression models that reflect the difference in neonatal metabolite concentrations in SDS for an interquartile range increase in maternal total bisphenol concentration (in μmol/g creatinine). Model includes gestational age at intake, maternal age, parity, education, pre-pregnancy body mass index, smoking habits, alcohol consumption and maternal kcal intake

*p-value < 0.05

### Maternal bisphenol A concentrations and neonatal metabolite profiles

Higher maternal average BPA concentrations were associated with higher neonatal mono-unsaturated Lyso.PC.e concentrations (difference 0.20 SDS (95% CI 0.04–0.35) per IQR increase in BPA) (Table 3). Trimester-specific analyses showed that specifically higher maternal second trimester BPA concentrations were associated with higher neonatal mono-unsaturated Lyso.PC.e concentrations (p-value < 0.05). Of the individual metabolites, higher average and second trimester BPA concentrations were associated with higher neonatal Lyso.PC.e C18:1 concentrations (p-value < 0.05) (Supplementary Table S9).

**Table 3:** Associations of maternal average and trimester-specific bisphenol A exposure in pregnancy with cord blood metabolic groups and subgroups

Neonatal metabolite group	Maternal average bisphenol A		Maternal first trimester bisphenol A		Maternal second trimester bisphenol A		Maternal third trimester bisphenol A	
	Difference in neonatal metabolite group (SDS) (95% CI)	P-value	Difference in neonatal metabolite group (SDS) (95% CI)	P-value	Difference in neonatal metabolite group (SDS) (95% CI)	P-value	Difference in neonatal metabolite group (SDS) (95% CI)	P-value
Amino acids (AA)	− 0.06 (− 0.22, 0.10)	0.464	− 0.03 (− 0.20, 0.13)	0.716	− 0.09 (− 0.26, 0.08)	0.285	− 0.03 (− 0.18, 0.13)	0.750
Branched-chain AA	− 0.09 (− 0.24, 0.07)	0.269	− 0.06 (− 0.22, 0.10)	0.442	− 0.08 (− 0.25, 0.09)	0.361	0.02 (− 0.14, 0.17)	0.840
Aromatic AA	− 0.03 (− 0.18, 0.13)	0.745	− 0.02 (− 0.19, 0.14)	0.787	− 0.07 (− 0.24, 0.10)	0.429	0.05 (− 0.11, 0.20)	0.536
Essential AA	− 0.03 (− 0.19, 0.12)	0.681	− 0.04 (− 0.20, 0.12)	0.606	− 0.03 (− 0.20, 0.14)	0.725	0.01 (− 0.14, 0.16)	0.897
Non-essential AA	− 0.07 (− 0.22, 0.09)	0.400	− 0.02 (− 0.19, 0.15)	0.809	− 0.12 (− 0.29, 0.05)	0.174	− 0.05 (− 0.20, 0.11)	0.572
Non-esterified fatty acids (NEFA)	− 0.10 (− 0.25, 0.05)	0.199	− 0.16 (− 0.32, 0.00)	0.046*	0.01 (− 0.16, 0.17)	0.942	0.08 (− 0.07, 0.23)	0.311
Saturated NEFA	− 0.10 (− 0.24, 0.06)	0.252	− 0.17 (− 0.33, − 0.01)	0.039*	0.02 (− 0.15, 0.19)	0.813	0.09 (− 0.06, 0.24)	0.248
Mono-unsaturated NEFA	− 0.10 (− 0.25, 0.05)	0.196	− 0.14 (− 0.29, 0.02)	0.097	0.00 (− 0.17, 0.16)	0.966	0.05 (− 0.10, 0.21)	0.488
Poly-unsaturated NEFA	− 0.11 (− 0.26, 0.05)	0.184	− 0.17 (− 0.33, − 0.01)	0.040*	0.00 (− 0.18, 0.17)	0.967	0.09 (− 0.07, 0.24)	0.281
Diacyl-phosphatidylcholines (PC.aa)	− 0.09 (− 0.07, 0.24)	0.265	0.036 (− 0.13, 0.20)	0.664	0.06 (− 0.11, 0.22)	0.517	0.04 (− 0.12, 0.19)	0.642
Saturated PC.aa	0.12 (− 0.04, 0.27)	0.129	0.06 (− 0.10, 0.22)	0.486	0.12 (− 0.05, 0.29)	0.163	0.04 (− 0.11, 0.19)	0.600
Mono-unsaturated PC.aa	0.14 (− 0.02, 0.29)	0.085	0.06 (− 0.10, 0.23)	0.452	0.14 (− 0.03, 0.31)	0.112	0.01 (− 0.14, 0.17)	0.865
Poly-unsaturated PC.aa	0.07 (− 0.08, 0.23)	0.357	0.03 (− 0.13, 0.19)	0.725	0.03 (− 0.14, 0.20)	0.699	0.04 (− 0.11, 0.19)	0.626
Acyl-alkyl-phosphatidylcholines (PC.ae)	0.07 (− 0.09, 0.22)	0.389	0.03 (− 0.13, 0.20)	0.708	0.04 (− 0.13, 0.21)	0.652	0.06 (− 0.09, 0.21)	0.432
Saturated PC.ae	0.03 (− 0.13, 0.18)	0.711	− 0.02 (− 0.18, 0.14)	0.801	0.10 (− 0.07, 0.27)	0.238	0.04 (− 0.11, 0.19)	0.583
Mono-unsaturated PC.ae	0.11 (− 0.04, 0.27)	0.152	0.03 (− 0.14, 0.19)	0.736	0.14 (− 0.02, 0.31)	0.092	0.03 (− 0.13, 0.18)	0.719
Poly-unsaturated PC.ae	0.07 (− 0.09, 0.22)	0.411	0.04 (− 0.12, 0.21)	0.619	0.01 (− 0.16, 0.17)	0.946	0.07 (− 0.09, 0.22)	0.392
Acyl-lysophosphatidylcholines (Lyso.PC.a)	0.06 (− 0.09, 0.22)	0.422	− 0.02 (− 0.19, 0.14)	0.795	0.05 (− 0.12, 0.22)	0.571	0.03 (− 0.12, 0.19)	0.678
Saturated Lyso.PC.a	0.07 (− 0.09, 0.23)	0.891	− 0.03 (− 0.19, 0.14)	0.729	0.02 (− 0.15, 0.19)	0.840	0.07 (− 0.09, 0.22)	0.400
Mono-unsaturated Lyso.PC.a	0.05 (− 0.11, 0.21)	0.525	− 0.01 (− 0.17, 0.15)	0.916	0.10 (− 0.07, 0.27)	0.245	− 0.05 (− 0.21, 0.10)	0.491
Poly-unsaturated Lyso.PC.a	0.04 (− 0.12, 0.19)	0.625	0.00 (− 0.16, 0.16)	0.989	0.09 (− 0.07, 0.26)	0.263	− 0.01 (− 0.16, 0.14)	0.876
Alkyl-lysophosphatidylcholines (Lyso.PC.e)	0.04 (− 0.11, 0.20)	0.579	0.04 (− 0.12, 0.20)	0.638	0.09 (− 0.08, 0.26)	0.287	0.00 (− 0.15, 0.15)	1.000
Saturated Lyso.PC.e	0.01 (− 0.15, 0.17)	0.898	0.02 (− 0.15, 0.18)	0.840	0.06 (− 0.11, 0.23)	0.491	− 0.02 (− 0.17, 0.13)	0.809
Mono-unsaturated Lyso.PC.e	0.20 (0.04, 0.35)	0.011*	0.14 (− 0.02, 0.30)	0.082	0.21 (0.05, 0.37)	0.012*	0.08 (− 0.07, 0.23)	0.290
Sphingomyelines (SM)	0.11 (− 0.04, 0.27)	0.146	0.04 (− 0.14, 0.20)	0.656	0.12 (− 0.04, 0.29)	0.148	0.07 (− 0.08, 0.22)	0.377
Mono-unsaturated SM	0.10 (− 0.06, 0.25)	0.216	0.01 (− 0.15, 0.17)	0.920	0.15 (− 0.02, 0.32)	0.075	0.06 (− 0.10, 0.21)	0.464
Poly-unsaturated SM	0.12 (− 0.03, 0.28)	0.117	0.06 (− 0.10, 0.22)	0.471	0.09 (− 0.07, 0.26)	0.267	0.07 (− 0.08, 0.23)	0.335
Free carnitine	− 0.02 (− 0.18, 0.14)	0.798	− 0.02 (− 0.18, 0.15)	0.858	− 0.01 (− 0.18, 0.16)	0.885	− 0.04 (− 0.19, 0.12)	0.659
Acyl-carnitines (Carn.a)	− 0.04 (− 0.19, 0.12)	0.656	− 0.08 (− 0.24, 0.08)	0.323	0.02 (− 0.15, 0.18)	0.860	− 0.03 (− 0.12, 0.18)	0.727
Small-chain Carn.a	− 0.03 (− 0.20, 0.10)	0.496	− 0.10 (− 0.26, 0.06)	0.236	0.01 (− 0.15, 0.18)	0.897	0.02 (− 0.13, 0.17)	0.765
Medium-chain Carn.a	− 0.05 (− 0.10, 0.20)	0.541	0.02 (− 0.13, 0.18)	0.769	0.06 (− 0.10, 0.22)	0.478	− 0.03 (− 0.18, 0.12)	0.712
Large-chain Carn.a	0.06 (− 0.10, 0.21)	0.489	0.00 (− 0.16, 0.16)	0.985	− 0.01 (− 0.16, 0.18)	0.897	0.07 (− 0.08, 0.22)	0.365

Values represent regression coefficients (95% confidence interval) and corresponding p-values from linear regression models that reflect the difference in neonatal metabolite concentrations in SDS for an interquartile range increase in maternal bisphenol A concentration (in μmol/g creatinine). Model includes gestational age at intake, maternal age, parity, education, pre-pregnancy body mass index, smoking habits, alcohol consumption and maternal kcal intake

*p-value < 0.05

Higher maternal first trimester BPA concentrations were associated with lower overall neonatal NEFA levels, saturated NEFA levels and poly-unsaturated NEFA levels (all p-values < 0.05). The strongest associations were present for NEFA C20:5 and NEFA C24:5 (Supplementary Table S10). All associations were not explained by birth characteristics (results not shown)**.** When we considered multiple testing, all associations disappeared. No associations were found of maternal average and third-trimester BPA concentrations with other neonatal metabolite groups.

### Maternal bisphenol S concentrations and neonatal metabolite profiles

Higher maternal average, but not trimester-specific, BPS concentrations were associated with lower neonatal overall Lyso.PC.e and saturated Lyso.PC.e concentrations (differences − 0.20 SDS (95% CI − 0.39 to − 0.01); − 0.21 SDS (95% CI − 0.40 to − 0.02) per IQR increase in BPS respectively) (Table 4). Of the individual metabolites, higher average maternal BPS concentrations were associated with lower neonatal Lyso.PC.e C18:0 concentrations (p-value < 0.05) (Supplementary Table S11).

**Table 4:** Associations of maternal average and trimester-specific bisphenol S exposure in pregnancy with cord blood metabolic groups and subgroups

Neonatal metabolite group	Maternal average bisphenol S		Maternal first trimester bisphenol S		Maternal second trimester bisphenol S		Maternal third trimester bisphenol S	
	Difference in neonatal metabolite group (SDS) (95% CI)	P-value	Difference in neonatal metabolite group (SDS) (95% CI)	P-value	Difference in neonatal metabolite group (SDS) (95% CI)	P-value	Difference in neonatal metabolite group (SDS) (95% CI)	P-value
Amino acids (AA)	− 0.12 (− 0.31, 0.08)	0.232	− 0.04 (− 0.24, 0.16)	0.689	− 0.13 (− 0.28, 0.03)	0.105	− 0.09 (− 0.20, 0.02)	0.122
Branched-chain AA	− 0.15 (− 0.34, 0.04)	0.120	− 0.13 (− 0.33, 0.07)	0.190	− 0.11 (− 0.27, 0.04)	0.149	− 0.04 (− 0.15, 0.07)	0.444
Aromatic AA	− 0.13 (− 0.33, 0.06)	0.184	− 0.12 (− 0.32, 0.08)	0.233	− 0.14 (− 0.29, 0.02)	0.081	− 0.07 (− 0.18, 0.05)	0.241
Essential AA	− 0.13 (− 0.32, 0.06)	0.180	− 0.10 (− 0.29, 0.10)	0.341	− 0.10 (− 0.25, 0.05)	0.202	− 0.08 (− 0.19, 0.03)	0.138
Non-essential AA	− 0.10 (− 0.30, 0.10)	0.315	− 0.01 (− 0.21, 0.20)	0.958	− 0.13 (− 0.29, 0.02)	0.097	− 0.08 (− 0.19, 0.03)	0.151
Non-esterified fatty acids (NEFA)	− 0.02 (− 0.21, 0.18)	0.869	0.13 (− 0.07, 0.32)	0.213	− 0.07 (− 0.23, 0.08)	0.354	− 0.11 (− 0.22, 0.00)	0.050*
Saturated NEFA	− 0.05 (− 0.24, 0.14)	0.575	0.10 (− 0.09, 0.30)	0.303	− 0.08 (− 0.23, 0.08)	0.331	− 0.11 (− 0.22, − 0.01)	0.038*
Mono-unsaturated NEFA	0.01 (− 0.18, 0.20)	0.951	0.14 (− 0.06, 0.33)	0.170	− 0.09 (− 0.24, 0.06)	0.247	− 0.09 (− 0.20, 0.02)	0.096
Poly-unsaturated NEFA	0.04 (− 0.16, 0.24)	0.692	0.15 (− 0.06, 0.34)	0.170	− 0.03 (− 0.19, 0.13)	0.701	− 0.11 (− 0.22, 0.01)	0.063
Diacyl-phosphatidylcholines (PC.aa)	− 0.01 (− 0.20, 0.18)	0.904	0.03 (− 0.17, 0.23)	0.778	− 0.06 (− 0.21, 0.10)	0.470	− 0.07 (− 0.18, 0.04)	0.204
Saturated PC.aa	− 0.04 (− 0.23, 0.15)	0.698	− 0.04 (− 0.23, 0.16)	0.726	0.03 (− 0.13, 0.18)	0.712	− 0.07 (− 0.18, 0.04)	0.224
Mono-unsaturated PC.aa	− 0.01 (− 0.20, 0.19)	0.926	0.06 (− 0.14, 0.26)	0.583	− 0.04 (− 0.19, 0.12)	0.650	− 0.10 (− 0.21, 0.01)	0.066
Poly-unsaturated PC.aa	− 0.01 (− 0.20, 0.18)	0.918	0.02 (− 0.17, 0.22)	0.808	− 0.06 (− 0.22, 0.09)	0.415	− 0.06 (− 0.17, 0.05)	0.279
Acyl-alkyl-phosphatidylcholines (PC.ae)	− 0.09 (− 0.28, 0.10)	0.340	− 0.09 (− 0.28, 0.11)	0.389	− 0.03 (− 0.18, 0.12)	0.686	− 0.04 (− 0.15, 0.07)	0.438
Saturated PC.ae	− 0.16 (− 0.35, 0.03)	0.099	− 0.15 (− 0.35, 0.05)	0.131	− 0.08 (− 0.22,0.09)	0.399	− 0.08 (− 0.18, 0.03)	0.175
Mono-unsaturated PC.ae	− 0.12 (− 0.31, 0.08)	0.237	− 0.12 (− 0.31, 0.08)	0.252	0.03 (− 0.12, 0.19)	0.663	− 0.08 (− 0.19, 0.02)	0.128
Poly-unsaturated PC.ae	− 0.07 (− 0.26, 0.12)	0.487	− 0.06 (− 0.26, 0.14)	0.537	− 0.03 (− 0.09, 0.12)	0.664	− 0.03 (− 0.14, 0.08)	0.649
Acyl-lysophosphatidylcholines (Lyso.PC.a)	− 0.04 (− 0.24, 0.15)	0.653	0.01 (− 0.19, 0.21)	0.953	− 0.13 (− 0.28, 0.03)	0.102	− 0.08 (− 0.19, 0.03)	0.154
Saturated Lyso.PC.a	− 0.02 (− 0.22, 0.17)	0.832	0.01 (− 0.19, 0.21)	0.937	− 0.16 (− 0.31, − 0.01)	0.043*	− 0.05 (− 0.16, 0.06)	0.390
Mono-unsaturated Lyso.PC.a	− 0.09 (− 0.28, 0.10)	0.348	0.00 (− 0.20, 0.20)	0.992	− 0.03 (− 0.19, 0.12)	0.688	− 0.13 (− 0.24, − 0.02)	0.017*
Poly-unsaturated Lyso.PC.a	− 0.06 (− 0.25, 0.13)	0.540	0.01 (− 0.19, 0.20)	0.963	− 0.08 (− 0.22, 0.08)	0.351	− 0.10 (− 0.21, 0.01)	0.072
Alkyl-lysophosphatidylcholines (Lyso.PC.e)	− 0.20 (− 0.39, − 0.01)	0.037*	− 0.11 (− 0.31, 0.08)	0.256	− 0.12 (− 0.28, 0.03)	0.114	− 0.03 (− 0.14, 0.08)	0.564
Saturated Lyso.PC.e	− 0.21 (− 0.40, − 0.02)	0.030*	− 0.13 (− 0.33, 0.07)	0.193	− 0.13 (− 0.28, 0.02)	0.090	− 0.03 (− 0.14, 0.08)	0.632
Mono-unsaturated Lyso.PC.e	− 0.07 (− 0.25, 0.12)	0.496	0.01 (− 0.18, 0.21)	0.891	− 0.02 (− 0.18, 0.14)	0.768	− 0.05 (− 0.15, 0.06)	0.415
Sphingomyelines (SM)	− 0.04 (− 0.23, 0.15)	0.704	− 0.02 (− 0.22, 0.18)	0.834	− 0.02 (− 0.17, 0.14)	0.844	− 0.07 (− 0.18, 0.03)	0.181
Mono-unsaturated SM	− 0.10 (− 0.29, 0.09)	0.295	− 0.10 (− 0.30, 0.10)	0.300	− 0.01 (− 0.16, 0.15)	0.934	− 0.06 (− 0.17, 0.05)	0.272
Poly-unsaturated SM	0.02 (− 0.17, 0.21)	0.828	0.05 (− 0.15, 0.35)	0.614	− 0.02 (− 0.17, 0.13)	0.777	− 0.08 (− 0.19, 0.03)	0.142
Free carnitine	0.06 (− 0.14, 0.25)	0.577	0.15 (− 0.05, 0.35)	0.152	− 0.03 (− 0.18, 0.14)	0.746	− 0.10 (− 0.21, 0.01)	0.081
Acyl-carnitines (Carn.a)	0.03 (− 0.15, 0.22)	0.777	0.22 (0.02, 0.41)	0.028*	− 0.03 (− 0.18, 0.12)	0.676	− 0.12 (− 0.22, − 0.01)	0.033*
Small-chain Carn.a	0.02 (− 0.17, 0.20)	0.862	0.19 (0.00, 0.39)	0.049*	− 0.03 (− 0.18, 0.12)	0.690	− 0.11 (− 0.22, 0.00)	0.047*
Medium-chain Carn.a	0.08 (− 0.11, 0.27)	0.405	0.20 (0.00, 0.29)	0.046*	0.04 (− 0.11, 0.20)	0.592	− 0.07 (− 0.18, 0.04)	0.206
Large-chain Carn.a	0.07 (− 0.13, 0.26)	0.509	0.28 (0.08, 0.47)	0.006*	− 0.07 (− 0.22, 0.08)	0.376	− 0.13 (− 0.24, − 0.03)	0.016*

Values represent regression coefficients (95% confidence interval) and corresponding p-values from linear regression models that reflect the difference in neonatal metabolite concentrations in SDS for an interquartile range increase in maternal bisphenol S concentration (in μmol/g creatinine). Model includes gestational age at intake, maternal age, parity, education, pre-pregnancy body mass index, smoking habits, alcohol consumption and maternal kcal intake

*p-value < 0.05

Trimester-specific analyses showed that higher maternal first trimester BPS concentrations were associated with higher neonatal overall Carn.a, and small-, medium- and large-chain Carn.a concentrations (all p-values < 0.05). Strongest effects were present for the individual metabolites Carn.a C16:0.Oxo, Carn.a C18:2.OH, Carn.a C20:0, Carn.a C20:3 and Carn.a C20:4 (Supplementary Table S12). Contrary, higher maternal third trimester BPS concentrations were associated with lower levels of neonatal overall Carn.a, small-chain Carn.a, long-chain Carn.a, overall NEFA, saturated NEFA and mono-unsaturated Lyso.PC.a concentrations (all p-values < 0.05). Associations were present with 11 individual neonatal Carn.a metabolites and 5 NEFA metabolites (Supplementary Tables S12 and 13). Overall, these associations were not explained by birth characteristics and tended to remain after using FDR-correction for multiple testing, but not after Bonferroni-correction (results not shown). No associations between maternal overall or trimester-specific BPS concentrations with neonatal AA, PC.aa, PC.ae or SM were present.

### Maternal bisphenol F concentrations and neonatal metabolite profiles

Maternal average BPF exposure was not available as second trimester BPF did not meet the criteria for inclusion. Higher maternal first trimester BPF concentrations were associated with higher neonatal medium-chain Carn.a levels only (p-value < 0.05) (Table 5). Higher maternal third trimester BPF concentrations were associated with lower neonatal saturated NEFA, mono-unsaturated PC.ae, mono-unsaturated Lyso.PC.a, overall PC.aa, saturated PC.aa, mono- and poly-unsaturated PC.aa, overall SM and mono- and poly-unsaturated SM concentrations (all p-values < 0.05). The strongest associations were present for PC.aa.C30.3, PC.aa.C34.5, PC.aa.C36.1, PC.aa.C36.2 and SM.a.C42.4 (Supplementary Tables S14 and 15). Overall, associations were not explained by birth characteristics, and tended to remain significant after FDR correction, but not after Bonferroni-correction (results not shown). No associations of maternal BPF concentrations with neonatal AA, lyso.PC.ae, free Carn and Carn.a were present.

**Table 5:** Associations of maternal trimester-specific bisphenol F exposure in pregnancy with cord blood metabolic groups and subgroups

Neonatal metabolite group	Maternal first trimester bisphenol F		Maternal third trimester bisphenol F	
	Difference in neonatal metabolite group (SDS) (95% CI)	P-value	Difference in neonatal metabolite group (SDS) (95% CI)	P-value
Amino acids (AA)	0.02 (− 0.18, 0.22)	0.836	− 0.04 (− 0.22, 0.14)	0.635
Branched-chain AA	0.00 (− 0.19, 0.19)	0.999	0.00 (− 0.27, 0.28)	0.985
Aromatic AA	0.02 (− 0.18, 0.21)	0.883	− 0.12 (− 0.29, 0.06)	0.193
Essential AA	0.00 (− 0.19, 0.19)	0.998	− 0.09 (− 0.26, 0.09)	0.326
Non-essential AA	0.03 (− 0.17, 0.23)	0.760	− 0.01 (− 0.19, 0.17)	0.885
Non-esterified fatty acids (NEFA)	0.03 (− 0.16, 0.22)	0.756	− 0.17 (− 0.34, 0.01)	0.056
Saturated NEFA	0.03 (− 0.16, 0.22)	0.732	− 0.18 (− 0.35, − 0.01)	0.043*
Mono-unsaturated NEFA	0.05 (− 0.14, 0.24)	0.628	− 0.15 (− 0.32, 0.03)	0.094
Poly-unsaturated NEFA	− 0.01 (− 0.20, 0.19)	0.947	− 0.16 (− 0.34, 0.02)	0.082
Diacyl-phosphatidylcholines (PC.aa)	0.02 (− 0.16, 0.22)	0.777	− 0.21 (− 0.38, − 0.03)	0.019*
Saturated PC.aa	− 0.02 (− 0.22, 0.17)	0.825	− 0.20 (− 0.37, − 0.02)	0.027*
Mono-unsaturated PC.aa	0.05 (− 0.15, 0.25)	0.612	− 0.23 (− 0.41, − 0.06)	0.009*
Poly-unsaturated PC.aa	0.02 (− 0.17, 0.21)	0.810	− 0.19 (− 0.36, − 0.02)	0.028*
Acyl-alkyl-phosphatidylcholines (PC.ae)	− 0.07 (− 0.27, 0.12)	0.452	− 0.16 (− 0.34, 0.01)	0.065
Saturated PC.ae	− 0.12 (− 0.31, 0.07)	0.199	− 0.19 (− 0.37, − 0.02)	0.026*
Mono-unsaturated PC.ae	− 0.04 (− 0.23, 0.15)	0.699	− 0.22 (− 0.39, − 0.05)	0.011*
Poly-unsaturated PC.ae	− 0.07 (− 0.26, 0.13)	0.511	− 0.13 (− 0.31, 0.04)	0.131
Acyl-lysophosphatidylcholines (Lyso.PC.a)	0.08 (− 0.11, 0.27)	0.420	− 0.11 (− 0.29, 0.07)	0.220
Saturated Lyso.PC.a	0.10 (− 0.10, 0.30)	0.320	− 0.06 (− 0.24, 0.12)	0.482
Mono-unsaturated Lyso.PC.a	− 0.01 (− 0.21, 0.18)	0.895	− 0.19 (− 0.36, − 0.01)	0.035*
Poly-unsaturated Lyso.PC.a	0.06 (− 0.13, 0.25)	0.562	− 0.15 (− 0.32, 0.02)	0.086
Alkyl-lysophosphatidylcholines (Lyso.PC.e)	− 0.05 (− 0.25, 0.14)	0.585	− 0.12 (− 0.30, 0.05)	0.158
Saturated Lyso.PC.e	− 0.05 (− 0.24, 0.14)	0.598	− 0.13 (− 0.30, 0.05)	0.158
Mono-unsaturated Lyso.PC.e	− 0.03 (− 0.22, 0.16)	0.732	− 0.09 (− 0.26, 0.08)	0.305
Sphingomyelines (SM)	− 0.01 (− 0.20, 0.18)	0.910	− 0.19 (− 0.37, − 0.03)	0.026*
Mono-unsaturated SM	− 0.05 (− 0.24, 0.15)	0.643	− 0.17 (− 0.35, 0.00)	0.048*
Poly-unsaturated SM	0.02 (− 0.17, 0.21)	0.850	− 0.20 (− 0.37, − 0.03)	0.019*
Free carnitine	0.09 (− 0.10, 0.29)	0.350	− 0.09 (− 0.27, 0.08)	0.299
Acyl-carnitines (Carn.a)	0.07 (− 0.12, 0.26)	0.451	− 0.12 (− 0.29, 0.06)	0.188
Small-chain Carn.a	0.05 (− 0.15, 0.24)	0.643	− 0.11 (− 0.28, 0.06)	0.206
Medium-chain Carn.a	0.21 (0.02, 0.39)	0.030*	− 0.07 (− 0.24, 0.10)	0.445
Large-chain Carn.a	0.14 (− 0.05, 0.34)	0.146	− 0.11 (− 0.29, 0.06)	0.204

Values represent regression coefficients (95% confidence interval) and corresponding p-values from linear regression models that reflect the difference in neonatal metabolite concentrations in SDS for an interquartile range increase in maternal bisphenol F concentration (in μmol/g creatinine). Model includes gestational age at intake, maternal age, parity, education, pre-pregnancy body mass index, smoking habits, alcohol consumption and maternal kcal intake. Second trimester maternal bisphenol F concentrations did not meet the criteria for inclusion (≥ 20% of concentrations above limit of detection). Therefore, no maternal average bisphenol F concentrations were calculated

*p-value < 0.05

## Discussion

### Main findings

In a population-based prospective cohort study, higher maternal bisphenol A, F and S concentrations in pregnancy were associated with alterations in the neonatal metabolite profile, mainly in NEFA, PL and acyl-carnitines concentrations. The strongest associations were present for maternal third trimester bisphenol F and S exposure with phosphatidylcholine, sphingomyelins and acyl-carnitines. These associations were not explained by maternal socio-demographic and lifestyle characteristics or birth characteristics. No associations of maternal grouped bisphenol exposure with neonatal metabolite profiles were present.

### Interpretation of findings

The endocrine-disrupting chemicals BPA, BPS and BPF are among the most produced chemical compounds worldwide (Hormann et al., 2014; Liao & Kannan, 2014; Liao et al., 2012; Vandenberg et al., 2010). Observational studies have shown that higher maternal exposure to BPA, BPF and BPS in pregnancy is associated with altered fetal growth patterns and cardio-metabolic risk factors in the offspring (Ferguson et al., 2018; Harley et al., 2013; Hu et al., 2018, 2019; Lee et al., 2008, 2014; Philippat et al., 2014; Sol et al., 2020; Valvi et al., 2013). The mechanisms underlying these associations are not well-known but might involve alterations in neonatal metabolism.

Thus far, no studies among human populations assessed the influence of maternal bisphenol exposure in pregnancy on neonatal metabolite profiles. However, several animal studies have been performed which mainly suggest alterations in offspring lipid metabolism in response to maternal bisphenols exposure (Cabaton et al., 2013; Meng et al., 2019a, 2019b, 2019c). In a prospective study, maternal rats were exposed during pregnancy and lactation to BPA, BPS and BPF and offspring serum metabolomics were measured at 5 and 21 weeks of life (Meng et al., 2019a, 2019b, 2019c). Higher maternal BPA exposure was associated with alterations in offspring lipid metabolism, including lower Lyso.PC and PC in early life and higher NEFAs in later life. Higher maternal exposure BPS and BPF was associated with lower offspring NEFA levels in early life (Meng et al., 2019a, 2019b, 2019c). Another animal study showed that higher maternal BPS exposure was associated with higher offspring NEFA concentrations at 13 weeks of life (Meng et al., 2019a, 2019b, 2019c). Next to alterations in lipid metabolism, animal studies also showed that higher maternal BPA, BPS and BPF concentrations were associated with alterations in offspring AA, including valine, leucine and glutamine, and glycolytic and Krebs Cycle intermediates (Cabaton et al., 2013; Meng et al., 2019a, 2019b, 2019c).

Partly in line with these animal studies, we observed that higher maternal BPA and BPS concentrations were associated with lower neonatal Lyso.PC.e and NEFA concentrations (Cabaton et al., 2013; Meng et al., 2019a, 2019b, 2019c). Differences in observed associations in our study and animal studies of higher maternal bisphenol A and S concentrations with alterations in offspring NEFA concentrations might be related to the age of offspring at the time of measurement. In the previously mentioned animal studies, lower offspring NEFA concentrations were found in the neonatal period, while higher offspring NEFA concentrations were found in later life (Cabaton et al., 2013; Meng et al., 2019a, 2019b, 2019c). In addition, we observed that higher maternal first and third trimester BPS concentrations were strongly associated with neonatal Carn.a levels, and higher maternal third trimester BPF concentrations were strongly associated with lower neonatal SM and PC.aa levels. The changes in neonatal lipid metabolism are of interest, as several human studies have shown associations of lipid metabolism metabolites with altered fetal growth and body composition (Favretto et al., 2012; Hellmuth et al., 2017; Noto et al., 2016). Intra-uterine growth restricted newborns have higher NEFA, Lyso.PC and sphingosine concentrations, as compared to non-growth restricted newborns (Favretto et al., 2012; Hellmuth et al., 2017; Noto et al., 2016). Higher cord blood Carn levels in newborns have been associated with increased leptin and fat mass levels (Kadakia et al., 2018). Our observed associations were not explained by birth characteristics, which suggests that metabolic changes are independent from birthweight and gestational age at birth. Contrary to the animal studies, we observed no associations with neonatal AA metabolism (Cabaton et al., 2013; Meng et al., 2019a, 2019b, 2019c). Possibly these associations are more apparent at later ages, or after more extreme maternal exposure to bisphenols. We also observed no associations between total maternal bisphenol concentrations and neonatal metabolite levels. This might be explained by different and sometimes opposite effects of maternal exposure to the individual bisphenols A, S and F on neonatal metabolite profiles, leading to lack of associations after grouping of these bisphenols. Thus, our findings suggest that higher maternal exposure to bisphenol A, S and F in pregnancy is associated with alterations in neonatal lipid metabolism, but not with amino acid metabolism.

Fetal metabolite concentrations are the result of placental transfer and endogenous synthesis (Hivert et al., 2015). Because maternal bisphenol can freely cross the placenta, the observed changes in neonatal metabolite profile after higher bisphenol exposure could result from changes in both maternal and fetal metabolism (Nahar et al., 2015). We observed the strongest associations of higher maternal BPA, BPS and BPF concentrations with neonatal lipid metabolism in third trimester, possibly because this measurement is closest to birth. However, differences in trimester-specific effects might also be related to the stage of fetal development and the changes that occur in both maternal and fetal metabolism throughout pregnancy. The effects of higher maternal bisphenol concentrations on neonatal lipid metabolism might result from disruption of the steroidogenesis, as bisphenols cause alterations in estradiol, testosterone, progesterone and cortisol levels (Philips et al., 2017). Furthermore, higher maternal bisphenol exposure during pregnancy is associated changes in neonatal adiponectin and leptin levels in cord blood, which influences metabolic regulation and birth weight (Ashley-Martin et al., 2014; Chou et al., 2011; Minatoya et al., 2018). To obtain further insight into the role of alterations in maternal metabolite profiles, future studies should consider simultaneous repeated measurements of maternal plasma metabolites throughout pregnancy and cord blood metabolites at birth. Mediation analyses can aid in disentangling the potential underlying role of maternal metabolite alterations in response to bisphenol exposure and the subsequent influence on the metabolome of the neonate. Future studies are also needed to assess whether maternal and fetal hormonal changes in response to bisphenol exposure influence alterations in neonatal metabolism.

The effect estimates for the associations of higher maternal bisphenol concentrations with alterations in neonatal metabolite profile were small and partly lost significance after considering multiple testing, which may due to our small sample size. However, most associations remained after FDR-correction. Our findings should be considered hypothesis generating and are important from an etiological perspective, as they provide novel insight into potential mechanisms underlying the associations of maternal bisphenol exposure during pregnancy with adverse neonatal and child health outcomes. Our findings need to be replicated in further studies with larger numbers, more diverse populations and with a longer follow-up period.

### Methodological considerations

The main strength of the study is the prospective data collection from early pregnancy onwards, allowing repeated bisphenol measurements throughout the entire pregnancy. The bisphenol measurements and metabolomics analyses were only available in a subgroup for the Generation R Study, which consisted of Dutch, relatively high educated and healthy participants, as is also shown in the non-response analysis. This selected population might affect the generalizability of our findings. Due to the relatively small subgroup, we did not conduct analyses stratified by fetal sex or abnormal fetal growth as these sample sizes were small. Bisphenol concentrations in maternal urine were measured once per trimester. Due to the short biological half-life of bisphenols, one spot urine sample is possibly not representative for a whole trimester. We used a cut-off value for inclusion of bisphenols of ≥ 20% of samples above LOD. In bisphenols that met the inclusion criteria, we replaced compounds below LOD with the LOD divided by the square root of 2, which might have reduced variability in bisphenol exposures. We used a targeted metabolomic approach, allowing us to optimize the quantification of the metabolites of interest. However, relevant biological pathways might be missed. Further studies are needed using both untargeted and targeted metabolomics in larger and more generalizable study populations to replicate our findings and identify further novel pathways. Finally, we adjusted our analyses for many potential confounders. However, due to the observational nature of the study, residual confounding cannot be excluded.

## Conclusion

Higher maternal bisphenol A, S and F concentrations in pregnancy were associated with alterations in the neonatal metabolite profile, mainly in NEFA, PL and carnitines concentrations. The strongest associations were present for maternal third trimester bisphenol F and S exposure with neonatal phosphatidylcholines, sphingomyelins and carnitines. These associations were not explained by maternal socio-demographic and lifestyle characteristics or birth characteristics. Our findings should be considered hypothesis generating and are important from an etiological perspective, providing novel insight into potential mechanisms underlying the associations of maternal bisphenol exposure during pregnancy with adverse neonatal and child health outcomes.

## Supplementary Information

Below is the link to the electronic supplementary material.

Supplementary file1 (PDF 111 kb)

Supplementary file2 (DOCX 127 kb)

## Data Availability

The datasets generated and analyzed during the current study are not publicly available due to privacy restrictions but are available from the corresponding author on reasonable request.

## Abbreviations

AA: Amino Acids
AAA: Aromatic Amino Acids
BCAA: Branched-Chain Amino Acids
BPA: Bisphenol A
BPAF: Bisphenol AF
BPAP: Bisphenol AP
BPB: Bisphenol B
BPF: Bisphenol F
BPP: Bisphenol P
BPS: Bisphenol S
BPZ: Bisphenol Z
Carn: Carnitines
Carn.a: Acyl-Carnitines
CI: Confidence Interval
DAG: Directed Acyclic graph
Free Carn: Free Carnitine
HPLC: High-Performance Liquid Chromatography
IQR: Interquartile Range
LC/MS: Liquid Chromatography/Mass Spectrometry
LOD: Limit of Detection
Lyso.PC.a: Acyl-Lysophosphatidylcholines
Lyso.PC.e: Alkyl-Lysophosphatidylcholines
NEFA: Non-Esterified Fatty Acids
PC.aa: Diacyl-Phosphatidylcholines
PC.ae: Acyl-Alkyl-Phosphatidylcholines
PL: Phospholipids
SD: Standard Deviation
SDS: Standard Deviation Scores
SM: Sphingomyelins
QC: Quality Control
