# Supplementary figures and images for: Associations of maternal bisphenol urine concentrations during pregnancy with neonatal metabolomic profiles
Source: Metabolomics. 2021 Sep 13;17(9):84. doi: 10.1007/s11306-021-01836-w (PMC8437833; doi:10.1007/s11306-021-01836-w)

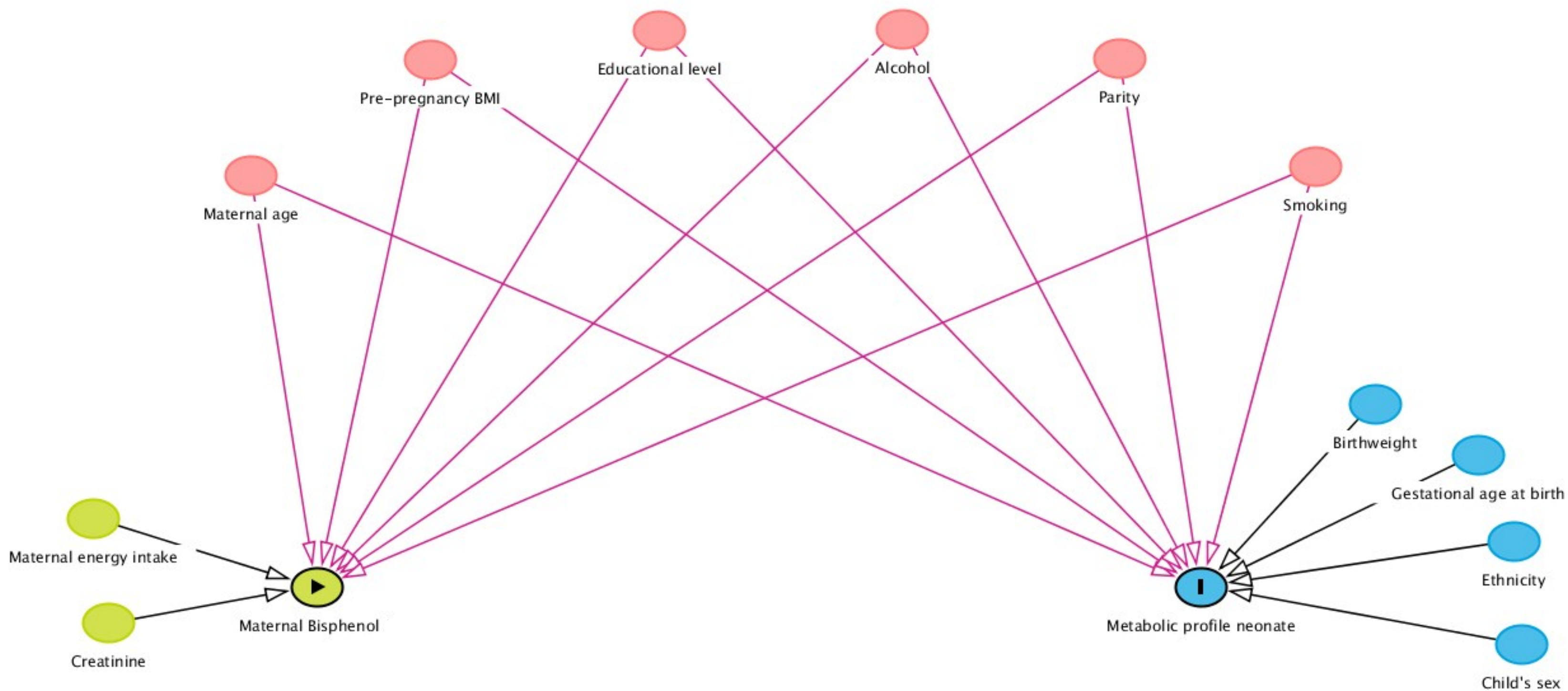

Supplement: Supplementary file 1 — Supplementary file1 (PDF 111 kb) [file 11306_2021_1836_MOESM1_ESM.pdf]
