# Supplementary material for: Associations of maternal bisphenol urine concentrations during pregnancy with neonatal metabolomic profiles
Source: Metabolomics. 2021 Sep 13;17(9):84. doi: 10.1007/s11306-021-01836-w (PMC8437833; doi:10.1007/s11306-021-01836-w)
Supplement: Supplementary file 2 — Supplementary file2 (DOCX 127 kb) [file 11306_2021_1836_MOESM2_ESM.docx]

**Supplementary Material**

**Associations of maternal bisphenol urine concentrations during pregnancy with neonatal metabolomic profiles**

Sophia M Blaauwendraad MD, Ellis Voerman MSc, Leonardo Trasande MD MPP, Kurunthachalam Kannan PhD, Susana Santos PhD, George JG Ruijter MD PhD, Chalana M Sol MD, Linda Marchioro PhD, Engy Shokry PhD, Berthold Koletzko MD PhD, Vincent WV Jaddoe MD PhD, Romy Gaillard MD PhD

**Corresponding author:** Romy Gaillard, MD, PhD. The Generation R Study Group (Na-29). Erasmus MC, University Medical Center, PO Box 2040, 3000 CA Rotterdam, the Netherlands. Email: [r.gaillard@erasmusmc.nl](mailto:r.gaillard@erasmusmc.nl)

**CONTENTS**

**Supplementary table S1.** Frequencies of urine samples with bisphenol concentrations above the limit of detection for the individual bisphenols

**Supplementary table S2.** Urine concentrations of bisphenols during pregnancy

**Supplementary table S3.** Parameters for mass-spectrometry detection and identification for amino-acids and non-esterified fatty acids, including the labelled internal standards .

**Supplementary table S4.** Parameters for mass-spectrometry detection and identifications for phospholipids and acyl-carnitines.

**Supplementary table S5.** Cord blood metabolite concentrations

**Supplementary table S6.** Comparison of general characteristics between mother-child pairs with bisphenol measurement and mother-child pairs without bisphenol measurement

**Supplementary table S7.** Cord blood metabolite concentrations in mother-child pairs with bisphenol measurement and mother-child pairs without bisphenol measurement

**Supplementary table S8.** Comparison of general characteristics between mother-child pairs with metabolite measurement and mother-child pairs without metabolite measurement

**Supplementary table S9.** Association of maternal average and second trimester bisphenol A urinary concentrations in pregnancy with cord blood alkyl-lysophosphatidylcholines.

**Supplementary table S10.** Association of maternal first trimester bisphenol A urinary concentrations in pregnancy with cord blood non-esterified fatty acids

**Supplementary table S11.** Association of maternal average bisphenol S urinary concentrations in pregnancy with cord blood metabolic alkyl-lysophosphatidylcholines

**Supplementary table S12.** Association of maternal first and third trimester bisphenol S urinary concentrations in pregnancy with cord blood acyl-carnitines

**Supplementary table S13.** Association of maternal third trimester bisphenol S urinary concentrations in pregnancy with cord blood non-esterified fatty acids

**Supplementary table S14.** Association of maternal third trimester bisphenol F urinary concentrations in pregnancy with cord blood diacyl-phosphatidylcholines

**Supplementary table S15.** Association of maternal third trimester bisphenol F urinary concentrations in pregnancy with cord blood sphingomyelins

**Supplementary figure S1.** Direct Acyclic Graph analysis on the hypnotized relationship between maternal bisphenol exposure, neonatal metabolites and the covariates.

**Supplementary table S1.** Frequencies of urine samples with bisphenol concentrations above the limit of detection for the individual bisphenols

| **Bisphenol** | **Level of detection**  ng/mL | **First trimester** | **Second trimester** | **Third trimester** |
| --- | --- | --- | --- | --- |
|  |  | Samples with bisphenol concentration above the limit of detection (%) | Samples with bisphenol concentration above the limit of detection (%) | Samples with bisphenol concentration above the limit of detection (%) |
| Bisphenol A | 0.15 | 80.4* | 93.8* | 93.3* |
| Bisphenol S | 0.05 | 70.7* | 31.1* | 20.0* |
| Bisphenol Z | 0.12 | 16.4 | 5.8 | 0.4 |
| Bisphenol B | 0.03 | 8.4 | 3.1 | 0.0 |
| Bisphenol F | 0.18 | 47.6* | 12.9 | 33.3* |
| Bisphenol AP | 0.07 | 12.0 | 0.0 | 0.0 |
| Bisphenol AF | 0.79 | 0.0 | 0.0 | 0.4 |
| Bisphenol P | 0.11 | 1.8 | 0.0 | 0.9 |
| *Meeting the inclusion criterion of ≥20% of samples above the limit of detection | | | | |

**Supplementary table S2.** Urine concentrations of bisphenols during pregnancy

|  | **First trimester**  Median (25th - 75th percentile) | **Second trimester**  Median (25th - 75th percentile) | **Third trimester**  Median (25th - 75th percentile) |
| --- | --- | --- | --- |
| Bisphenols (nmol/L) | 12.04 (4.94-28.08) | 5.99 (3.00-12.92) | 9.61 (4.82-17.02) |
| Bisphenol A (nmol/L) | 6.46 (1.51-15.57) | 5.48 (2.62-12.73) | 5.77 (2.90-10.36) |
| Bisphenol S (nmol/L) | 0.81 (0.23-3.88) | <LOD (<LOD-0.45) | <LOD (<LOD-0.13) |
| Bisphenol F (nmol/L) | <LOD (<LOD-3.68) | NA | <LOD (<LOD-3.43) |
| Values represented medians (25th – 75th percentiles) of absolute urine concentrations of grouped exposures and individual exposures (in nmol/L urine). Concentrations below limit detection (LOD) were imputed as LOD/√2. Only exposures with ≥20% of values above LOD are included. Others are non-applicable (NA). | | | |

**Supplementary table S3.** Parameters for mass-spectrometry detection and identification for amino-acids and non-esterified fatty acids, including the labelled internal standards .

| **ID*** | **Rt**  minutes | **Q1** | **Q3** | **DP** | **CE** | **CXP** | **Adduct** | **MSI ID Level** |
| --- | --- | --- | --- | --- | --- | --- | --- | --- |
| **Amino acids** | | | | | | |  |  |
| Ala1 | 7,6 | 146,182 | 44 | 11 | 25 | 4 | (M+H)+ | 1 |
| Ala2 | 7,6 | 146,182 | 90 | 11 | 13 | 12 | (M+H)+ | 1 |
| AlaIS | 7,6 | 150,168 | 48,1 | 31 | 25 | 6 | (M+H)+ | 1 |
| Arg1 | 7,3 | 231,201 | 70,1 | 21 | 39 | 8 | (M+H)+ | 1 |
| Arg2 | 7,3 | 231,276 | 60 | 21 | 33 | 8 | (M+H)+ | 1 |
| Arg3 | 7,3 | 231,276 | 172,2 | 21 | 21 | 8 | (M+H)+ | 1 |
| ArgIS | 7,3 | 236,201 | 75,1 | 21 | 39 | 8 | (M+H)+ | 1 |
| Asn1 | 5,4 | 189,303 | 144,1 | 21 | 17 | 6 | (M+H)+ | 1 |
| Asn2 | 5,4 | 189,303 | 74 | 21 | 27 | 8 | (M+H)+ | 1 |
| Asn3 | 5,4 | 189,303 | 130,3 | 21 | 19 | 4 | (M+H)+ | 1 |
| AsnIS | 5,4 | 191,116 | 145,2 | 21 | 19 | 6 | (M+H)+ | 1 |
| Asp1 | 13,8 | 246,262 | 144,3 | 21 | 19 | 6 | (M+H)+ | 1 |
| Asp2 | 13,8 | 246,262 | 88,1 | 21 | 27 | 2 | (M+H)+ | 1 |
| Asp3 | 13,8 | 246,262 | 74,2 | 21 | 35 | 8 | (M+H)+ | 1 |
| AspIS | 13,8 | 249,278 | 147,3 | 16 | 19 | 6 | (M+H)+ | 1 |
| Cit1 | 6,3 | 232,249 | 70,1 | 16 | 43 | 8 | (M+H)+ | 1 |
| Cit2 | 6,3 | 232,249 | 215,3 | 16 | 17 | 10 | (M+H)+ | 1 |
| Cit3 | 6,3 | 232,249 | 113,2 | 16 | 27 | 4 | (M+H)+ | 1 |
| CitIS | 6,3 | 234,237 | 115,2 | 16 | 27 | 4 | (M+H)+ | 1 |
| Cys1 | 11,8 | 353,133 | 129,9 | 21 | 29 | 14 | (M+H)+ | 1 |
| Cys2 | 11,8 | 353,078 | 73,9 | 21 | 47 | 8 | (M+H)+ | 1 |
| Cys3 | 11,8 | 353,078 | 208,1 | 21 | 21 | 8 | (M+H)+ | 1 |
| CysIS | 11,8 | 357,133 | 129,9 | 21 | 29 | 14 | (M+H)+ | 1 |
| Gln1 | 5,6 | 203,1 | 84,1 | 11 | 30 | 11 | (M+H)+ | 1 |
| Gln2 | 5,6 | 203,1 | 186,1 | 11 | 16 | 8 | (M+H)+ | 1 |
| Gln3 | 5,6 | 203,1 | 130,1 | 11 | 21 | 15 | (M+H)+ | 1 |
| GlnIS | 5,6 | 208,1 | 89,1 | 11 | 30 | 11 | (M+H)+ | 1 |
| Glu1 | 14,3 | 260,312 | 84 | 16 | 37 | 10 | (M+H)+ | 1 |
| Glu2 | 14,3 | 260,312 | 186,2 | 16 | 19 | 8 | (M+H)+ | 1 |
| Glu3 | 14,3 | 260,312 | 130,1 | 16 | 25 | 4 | (M+H)+ | 1 |
| GluIS | 14,3 | 263,297 | 87,1 | 16 | 35 | 10 | (M+H)+ | 1 |
| Gly1 | 6,2 | 132,19 | 76 | 16 | 13 | 8 | (M+H)+ | 1 |
| Gly2 | 6,2 | 132,19 | 57 | 16 | 19 | 6 | (M+H)+ | 1 |
| GlyIS | 6,2 | 134,17 | 77,9 | 16 | 13 | 10 | (M+H)+ | 1 |
| His1 | 6,8 | 212,18 | 109,9 | 16 | 27 | 14 | (M+H)+ | 1 |
| His2 | 6,8 | 212,271 | 83,1 | 21 | 41 | 10 | (M+H)+ | 1 |
| His3 | 6,8 | 212,271 | 93 | 21 | 41 | 8 | (M+H)+ | 1 |
| HisIS | 6,8 | 215,184 | 112,9 | 16 | 27 | 14 | (M+H)+ | 1 |
| Ile1 | 12,4 | 188,327 | 86 | 21 | 21 | 10 | (M+H)+ | 1 |
| Ile2 | 12,4 | 188,327 | 69 | 21 | 33 | 8 | (M+H)+ | 1 |
| Ile3 | 12,4 | 188,327 | 44,1 | 21 | 43 | 4 | (M+H)+ | 1 |
| Leu1 | 12,6 | 188,2 | 86 | 21 | 21 | 10 | (M+H)+ | 1 |
| Leu2 | 12,6 | 188,2 | 69 | 21 | 33 | 8 | (M+H)+ | 1 |
| Leu3 | 12,6 | 188,2 | 44,1 | 21 | 43 | 4 | (M+H)+ | 1 |
| LeuIS | 12,6 | 191,338 | 89,2 | 11 | 19 | 2 | (M+H)+ | 1 |
| Lys1 | 7,2 | 203,2 | 84,1 | 21 | 33 | 2 | (M+H)+ | 1 |
| Lys2 | 7,2 | 203,2 | 186,2 | 21 | 17 | 8 | (M+H)+ | 1 |
| Lys3 | 7,2 | 203,2 | 56 | 21 | 61 | 6 | (M+H)+ | 1 |
| LysIS | 7,2 | 207,2 | 88,1 | 21 | 33 | 2 | (M+H)+ | 1 |
| Met1 | 10,9 | 206,245 | 104,1 | 31 | 19 | 4 | (M+H)+ | 1 |
| Met2 | 10,9 | 206,245 | 61,1 | 31 | 41 | 6 | (M+H)+ | 1 |
| Met3 | 10,9 | 206,245 | 56 | 31 | 31 | 6 | (M+H)+ | 1 |
| MetIS | 10,9 | 209,2 | 107,1 | 11 | 30 | 5 | (M+H)+ | 1 |
| Orn1 | 6,7 | 189,304 | 70,1 | 16 | 29 | 8 | (M+H)+ | 1 |
| Orn2 | 6,7 | 189,304 | 172,2 | 16 | 15 | 8 | (M+H)+ | 1 |
| Orn3 | 6,7 | 189,304 | 116,1 | 16 | 21 | 6 | (M+H)+ | 1 |
| OrnIS | 6,7 | 191,338 | 174,1 | 11 | 15 | 8 | (M+H)+ | 1 |
| Phe1 | 12,8 | 222,248 | 120,3 | 21 | 23 | 4 | (M+H)+ | 1 |
| Phe2 | 12,8 | 222,248 | 103,1 | 21 | 49 | 10 | (M+H)+ | 1 |
| Phe3 | 12,8 | 222,248 | 77 | 21 | 69 | 8 | (M+H)+ | 1 |
| PheIS | 12,8 | 228,284 | 126,2 | 16 | 21 | 6 | (M+H)+ | 1 |
| Pro1 | 7,8 | 172,291 | 70,1 | 26 | 25 | 8 | (M+H)+ | 1 |
| Pro2 | 7,8 | 172,291 | 116,2 | 26 | 19 | 4 | (M+H)+ | 1 |
| Pro3 | 7,8 | 172,291 | 57,1 | 26 | 27 | 6 | (M+H)+ | 1 |
| ProIS | 7,8 | 175,18 | 73 | 16 | 27 | 8 | (M+H)+ | 1 |
| Pro2IS | 7,8 | 175,18 | 118,9 | 16 | 21 | 16 | (M+H)+ | 1 |
| Ser1 | 6 | 162,255 | 60 | 16 | 23 | 6 | (M+H)+ | 1 |
| Ser2 | 6 | 162,255 | 106,2 | 16 | 15 | 4 | (M+H)+ | 1 |
| Ser3 | 6 | 162,255 | 88,3 | 16 | 19 | 2 | (M+H)+ | 1 |
| SerIS | 6 | 165,255 | 63 | 16 | 23 | 6 | (M+H)+ | 1 |
| Thr1 | 7,2 | 176,24 | 73,9 | 16 | 23 | 10 | (M+H)+ | 1 |
| Thr2 | 7,2 | 176,24 | 55,9 | 16 | 31 | 6 | (M+H)+ | 1 |
| Thr3 | 7,2 | 176,24 | 102,1 | 16 | 19 | 4 | (M+H)+ | 1 |
| ThrIS | 7,2 | 180,24 | 75,9 | 16 | 23 | 10 | (M+H)+ | 1 |
| Trp1 | 13,3 | 261,284 | 244,2 | 21 | 17 | 10 | (M+H)+ | 1 |
| Trp2 | 13,3 | 261,284 | 159,3 | 21 | 25 | 6 | (M+H)+ | 1 |
| Trp3 | 13,3 | 261,284 | 132,4 | 21 | 41 | 6 | (M+H)+ | 1 |
| TrpIS | 13,3 | 266,284 | 249,2 | 21 | 17 | 10 | (M+H)+ | 1 |
| Tyr1 | 10,24 | 238,241 | 136,1 | 21 | 23 | 6 | (M+H)+ | 1 |
| Tyr2 | 10,24 | 238,241 | 91,2 | 21 | 47 | 4 | (M+H)+ | 1 |
| Tyr3 | 10,24 | 238,241 | 119,2 | 21 | 37 | 4 | (M+H)+ | 1 |
| TyrIS | 10,24 | 244,266 | 142,2 | 16 | 23 | 8 | (M+H)+ | 1 |
| Val1 | 10,8 | 174,213 | 72 | 16 | 19 | 8 | (M+H)+ | 1 |
| Val2 | 10,8 | 174,213 | 55 | 16 | 41 | 6 | (M+H)+ | 1 |
| Val3 | 10,8 | 174,213 | 118,2 | 16 | 15 | 6 | (M+H)+ | 1 |
| ValIS | 10,8 | 182,275 | 80,2 | 16 | 21 | 10 | (M+H)+ | 1 |
| **Non-esterified fatty acids** | | | | | | |  |  |
| 4_0 | 0,7 | 87 | 87 | -45 | -8 | -7 | (M-H)- | 1 |
| 5_0 | 0,8 | 101 | 101 | -45 | -8 | -7 | (M-H)- | 1 |
| 6_0 | 1,0 | 115,1 | 115,1 | -50 | -8 | -7 | (M-H)- | 1 |
| 7_0 | 1,2 | 129,1 | 129,1 | -50 | -8 | -7 | (M-H)- | 1 |
| 8_0 | 1,6 | 143,1 | 143,1 | -55 | -8 | -7 | (M-H)- | 1 |
| 9_0 | 2,0 | 157,1 | 157,1 | -55 | -8 | -7 | (M-H)- | 1 |
| 10_0 | 2,4 | 171,146 | 171,146 | -60 | -8 | -13 | (M-H)- | 1 |
| 11_0 | 2,7 | 185,162 | 185,162 | -65 | -8 | -11 | (M-H)- | 1 |
| 12_0 | 3,1 | 199,178 | 199,178 | -68 | -8 | -11 | (M-H)- | 1 |
| 12_1 | 2,6 | 197,162 | 197,162 | -72 | -8 | -7 | (M-H)- | 1 |
| 13_0 | 3,5 | 213,193 | 213,193 | -70 | -8 | -17 | (M-H)- | 1 |
| 13_1 | 2,9 | 211,178 | 211,178 | -74 | -8 | -7 | (M-H)- | 1 |
| 14_0 | 3,9 | 227,209 | 227,209 | -120 | -25 | -13 | (M-H)- | 1 |
| 14_1 | 3,3 | 225,193 | 225,193 | -75 | -8 | -13 | (M-H)- | 1 |
| 14_2 | 2,7 | 223,178 | 223,178 | -78 | -8 | -7 | (M-H)- | 1 |
| 15_0 | 4,2 | 241,225 | 241,225 | -75 | -8 | -7 | (M-H)- | 1 |
| 15_1 | 3,7 | 239,209 | 239,209 | -75 | -10 | -7 | (M-H)- | 1 |
| 16_0 | 4,6 | 255,24 | 255,24 | -150 | -35 | -13 | (M-H)- | 1 |
| 16_1 | 4,0 | 253,225 | 253,225 | -78 | -30 | -13 | (M-H)- | 1 |
| 16_2 | 3,5 | 251,209 | 251,209 | -79 | -8 | -7 | (M-H)- | 1 |
| 16_3 | 2,9 | 249,193 | 249,193 | -78 | -8 | -7 | (M-H)- | 1 |
| 16_4 | 2,4 | 247,178 | 247,178 | -78 | -8 | -7 | (M-H)- | 1 |
| 17_0 | 5,0 | 269,256 | 269,256 | -85 | -12 | -7 | (M-H)- | 1 |
| 17_1 | 4,4 | 267,24 | 267,24 | -75 | -10 | -7 | (M-H)- | 1 |
| 17_2 | 3,9 | 265,225 | 265,225 | -79 | -8 | -7 | (M-H)- | 1 |
| 18_0 | 5,4 | 283,272 | 283,272 | -150 | -35 | -7 | (M-H)- | 1 |
| 18_1 | 4,8 | 281,256 | 281,256 | -150 | -37 | -7 | (M-H)- | 1 |
| 18_2 | 4,2 | 279,24 | 279,24 | -130 | -32 | -7 | (M-H)- | 1 |
| 18_3 | 3,7 | 277,225 | 277,225 | -120 | -10 | -7 | (M-H)- | 1 |
| 18_4 | 3,1 | 275,209 | 275,209 | -72 | -8 | -7 | (M-H)- | 1 |
| 19_0 | 5,7 | 297,287 | 297,287 | -90 | -8 | -7 | (M-H)- | 1 |
| 19_1 | 5,2 | 295,272 | 295,272 | -85 | -8 | -7 | (M-H)- | 1 |
| 19_2 | 4,6 | 293,256 | 293,256 | -80 | -8 | -7 | (M-H)- | 1 |
| 20_0 | 6,1 | 311,303 | 311,303 | -95 | -8 | -13 | (M-H)- | 1 |
| 20_1 | 5,5 | 309,287 | 309,287 | -90 | -8 | -9 | (M-H)- | 1 |
| 20_2 | 5,0 | 307,272 | 307,272 | -85 | -8 | -9 | (M-H)- | 1 |
| 20_3 | 4,4 | 305,256 | 305,256 | -80 | -8 | -9 | (M-H)- | 1 |
| 20_4 | 3,9 | 303,24 | 303,24 | -150 | -10 | -9 | (M-H)- | 1 |
| 20_5 | 3,3 | 301,225 | 301,225 | -61 | -8 | -7 | (M-H)- | 1 |
| 22_0 | 6,8 | 339,334 | 339,334 | -100 | -14 | -11 | (M-H)- | 1 |
| 22_1 | 6,3 | 337,318 | 337,318 | -80 | -8 | -9 | (M-H)- | 1 |
| 22_2 | 5,7 | 335,303 | 335,303 | -80 | -8 | -11 | (M-H)- | 1 |
| 22_3 | 5,2 | 333,287 | 333,287 | -71 | -8 | -7 | (M-H)- | 1 |
| 22_4 | 4,6 | 331,272 | 331,272 | -62 | -8 | -7 | (M-H)- | 1 |
| 22_5 | 4,0 | 329,256 | 329,256 | -53 | -8 | -7 | (M-H)- | 1 |
| 22_6 | 3,5 | 327,24 | 327,24 | -150 | -6 | -13 | (M-H)- | 1 |
| 24_0 | 7,6 | 367,365 | 367,365 | -106 | -8 | -7 | (M-H)- | 1 |
| 24_1 | 7,0 | 365,35 | 365,35 | -97 | -8 | -11 | (M-H)- | 1 |
| 24_2 | 6,5 | 363,334 | 363,334 | -81 | -8 | -7 | (M-H)- | 1 |
| 24_3 | 5,9 | 361,318 | 361,318 | -69 | -8 | -7 | (M-H)- | 1 |
| 24_4 | 5,4 | 359,303 | 359,303 | -57 | -8 | -7 | (M-H)- | 1 |
| 24_5 | 4,8 | 357,287 | 357,287 | -44 | -8 | -7 | (M-H)- | 1 |
| 24_6 | 4,2 | 355,272 | 355,272 | -32 | -8 | -7 | (M-H)- | 1 |
| 26_0 | 8,3 | 395,397 | 395,397 | -113 | -8 | -7 | (M-H)- | 1 |
| 26_1 | 7,8 | 393,381 | 393,381 | -97 | -8 | -7 | (M-H)- | 1 |
| 26_2 | 7,2 | 391,365 | 391,365 | -82 | -8 | -7 | (M-H)- | 1 |
| 26_3 | 6,7 | 389,35 | 389,35 | -67 | -8 | -7 | (M-H)- | 1 |
| 26_4 | 6,1 | 387,334 | 387,334 | -52 | -8 | -7 | (M-H)- | 1 |
| 26_5 | 5,5 | 385,318 | 385,318 | -36 | -8 | -7 | (M-H)- | 1 |
| 26_6 | 5,0 | 383,303 | 383,303 | -21 | -8 | -7 | (M-H)- | 1 |
| 16_0-IS | 4,1 | 271 | 271 | -80 | -10 | -7 | (M-H)- | 1 |
| 20_4-IS | 3,7 | 311,24 | 311,24 | -70 | -10 | -9 | (M-H)- | 1 |
| 22_6-IS | 3,6 | 332,24 | 332,24 | -40 | -6 | -13 | (M-H)- | 1 |
| 22_0-IS | 5,9 | 342,33 | 342,33 | -100 | -14 | -11 | (M-H)- | 1 |
| 10_0-IS | 2,4 | 190,15 | 190,15 | -60 | -8 | -13 | (M-H)- | 1 |
| 6_0-IS | 0,9 | 126,1 | 126,1 | -50 | -8 | -7 | (M-H)- | 1 |
| ID metabolite identity, Rt retention time in minutes, Q1/Q3 quadrupole 1 and 3, IS internal standard, DP declustering potential, CE collison energy, CXP collision cell exit potential, MSI ID Metabolomics Standards Initiative identification.  *The numbers next to the ID refer to the different transitions used. | | | | | | | | |

**Supplementary table S4.** Parameters for mass-spectrometry detection and identifications for phospholipids and acyl-carnitines.

| **ID** | **Sofia.ID** | **Q1** | **Q3** | **CP1** | **CP2** | **Adduct** | **MSI ID Level** |  |  |  |  |  |  |  |
| --- | --- | --- | --- | --- | --- | --- | --- | --- | --- | --- | --- | --- | --- | --- |
| Carn.C0 | Carn | 162,1 | 85,1 | 29,25 | 29,85 | (M+H)+ | 1 |  |  |  |  |  |  |  |
| Carn.C10 | Carn.C10 | 316,2 | 85,1 | 0,1465 | 0,1395 | (M+H)+ | 1 |  |  |  |  |  |  |  |
| Carn.C10.1 | Carn.C10.1 | 314,2 | 85,1 | 0,13 | 0,122 | (M+H)+ | 1 |  |  |  |  |  |  |  |
| Carn.C10.2 | Carn.C10.2 | 312,2 | 85,1 | 0,0275 | 0,0275 | (M+H)+ | 1 |  |  |  |  |  |  |  |
| Carn.C12 | Carn.C12 | 344,3 | 85,1 | 0,06975 | 0,0705 | (M+H)+ | 1 |  |  |  |  |  |  |  |
| Carn.C12.1 | Carn.C12.1 | 342,3 | 85,1 | 0,13875 | 0,1245 | (M+H)+ | 1 |  |  |  |  |  |  |  |
| Carn.C12.DC | Carn.C12.DC | 374,3 | 85,1 | 0,0475 | 0,0495 | (M+H)+ | 1 |  |  |  |  |  |  |  |
| Carn.C14 | Carn.C14 | 372,3 | 85,1 | 0,03875 | 0,037 | (M+H)+ | 1 |  |  |  |  |  |  |  |
| Carn.C14.1 | Carn.C14.1 | 370,3 | 85,1 | 0,07725 | 0,078 | (M+H)+ | 1 |  |  |  |  |  |  |  |
| Carn.C14.1.OH | Carn.C14.1.OH | 386,3 | 85,1 | 0,0095 | 0,0095 | (M+H)+ | 1 |  |  |  |  |  |  |  |
| Carn.C14.2 | Carn.C14.2 | 368,3 | 85,1 | 0,014 | 0,0135 | (M+H)+ | 1 |  |  |  |  |  |  |  |
| Carn.C14.2.OH | Carn.C14.2.OH | 384,3 | 85,1 | 0,0075 | 0,007 | (M+H)+ | 1 |  |  |  |  |  |  |  |
| Carn.C16 | Carn.C16 | 400,3 | 85,1 | 0,075 | 0,0775 | (M+H)+ | 1 |  |  |  |  |  |  |  |
| Carn.C16.1 | Carn.C16.1 | 398,3 | 85,1 | 0,02625 | 0,027 | (M+H)+ | 1 |  |  |  |  |  |  |  |
| Carn.C16.1.OH | Carn.C16.1.OH | 414,3 | 85,1 | 0,01 | 0,0095 | (M+H)+ | 1 |  |  |  |  |  |  |  |
| Carn.C16.2 | Carn.C16.2 | 396,3 | 85,1 | 0,0045 | 0,005 | (M+H)+ | 1 |  |  |  |  |  |  |  |
| Carn.C16.2.OH | Carn.C16.2.OH | 412,3 | 85,1 | 0,01 | 0,0095 | (M+H)+ | 1 |  |  |  |  |  |  |  |
| Carn.C16.OH | Carn.C16.OH | 416,3 | 85,1 | 0,0055 | 0,0055 | (M+H)+ | 1 |  |  |  |  |  |  |  |
| Carn.C18 | Carn.C18 | 428,4 | 85,1 | 0,03425 | 0,0355 | (M+H)+ | 1 |  |  |  |  |  |  |  |
| Carn.C18.1 | Carn.C18.1 | 426,4 | 85,1 | 0,0915 | 0,0915 | (M+H)+ | 1 |  |  |  |  |  |  |  |
| Carn.C18.1.OH | Carn.C18.1.OH | 442,4 | 85,1 | 0,0075 | 0,0075 | (M+H)+ | 1 |  |  |  |  |  |  |  |
| Carn.C18.2 | Carn.C18.2 | 424,3 | 85,1 | 0,043 | 0,043 | (M+H)+ | 1 |  |  |  |  |  |  |  |
| Carn.C2 | Carn.C2 | 204,1 | 85,1 | 4,48 | 4,52 | (M+H)+ | 1 |  |  |  |  |  |  |  |
| Carn.C3 | Carn.C3 | 218,1 | 85,1 | 0,345 | 0,355 | (M+H)+ | 1 |  |  |  |  |  |  |  |
| Carn.C3.1 | Carn.C3.1 | 216,1 | 85,1 | 0,0055 | 0,005 | (M+H)+ | 1 |  |  |  |  |  |  |  |
| Carn.C3.DC.C4.OH. | Carn.C3.DC | 248,1 | 85,1 | 0,03875 | 0,0495 | (M+H)+ | 1 |  |  |  |  |  |  |  |
| Carn.C3.OH | Carn.C3.OH | 234,1 | 85,1 | 0,02125 | 0,022 | (M+H)+ | 1 |  |  |  |  |  |  |  |
| Carn.C4 | Carn.C4 | 232,2 | 85,1 | 0,179 | 0,1875 | (M+H)+ | 1 |  |  |  |  |  |  |  |
| Carn.C4.1 | Carn.C4.1 | 230,1 | 85,1 | 0,019 | 0,019 | (M+H)+ | 1 |  |  |  |  |  |  |  |
| Carn.C5 | Carn.C5 | 246,2 | 85,1 | 0,10375 | 0,103 | (M+H)+ | 1 |  |  |  |  |  |  |  |
| Carn.C5.1 | Carn.C5.1 | 244,2 | 85,1 | 0,0195 | 0,02 | (M+H)+ | 1 |  |  |  |  |  |  |  |
| Carn.C5.1.DC | Carn.C5.1.DC | 274,1 | 85,1 | 0,0175 | 0,015 | (M+H)+ | 1 |  |  |  |  |  |  |  |
| Carn.C5.DC.C6.OH. | Carn.C5.DC | 276,1 | 85,1 | 0,01925 | 0,0205 | (M+H)+ | 1 |  |  |  |  |  |  |  |
| Carn.C5.M.DC | Carn.C5.M.DC | 290,2 | 85,1 | 0,03225 | 0,031 | (M+H)+ | 1 |  |  |  |  |  |  |  |
| Carn.C5.OH.C3.DC.M. | Carn.C5.OH | 262,2 | 85,1 | 0,0575 | 0,0615 | (M+H)+ | 1 |  |  |  |  |  |  |  |
| Carn.C6.1 | Carn.C6.1 | 258,2 | 85,1 | 0,0125 | 0,0125 | (M+H)+ | 1 |  |  |  |  |  |  |  |
| Carn.C6.C4.1.DC. | Carn.C6 | 260,2 | 85,1 | 0,04475 | 0,046 | (M+H)+ | 1 |  |  |  |  |  |  |  |
| Carn.C7.DC | Carn.C7.DC | 304,2 | 85,1 | 0,02575 | 0,027 | (M+H)+ | 1 |  |  |  |  |  |  |  |
| Carn.C8 | Carn.C8 | 288,2 | 85,1 | 0,1105 | 0,1195 | (M+H)+ | 1 |  |  |  |  |  |  |  |
| Carn.C8.1 | Carn.C8.1 | 286,2 | 85,1 | 0,07525 | 0,0745 | (M+H)+ | 1 |  |  |  |  |  |  |  |
| Carn.C9 | Carn.C9 | 302,2 | 85,1 | 0,03425 | 0,034 | (M+H)+ | 1 |  |  |  |  |  |  |  |
| lysoPCaC14.0 | lyso.PC.a.C14.0 | 468,3 | 184 | 2,575 | 2,615 | (M+H)+ | 1 |  |  |  |  |  |  |  |
| lysoPCaC16.0 | lyso.PC.a.C16.0 | 496,3 | 184 | 98,775 | 95,6 | (M+H)+ | 1 |  |  |  |  |  |  |  |
| lysoPCaC16.1 | lyso.PC.a.C16.1 | 494,3 | 184 | 3 | 2,96 | (M+H)+ | 1 |  |  |  |  |  |  |  |
| lysoPCaC17.0 | lyso.PC.a.C17.0 | 510,4 | 184 | 1,7325 | 1,66 | (M+H)+ | 1 |  |  |  |  |  |  |  |
| lysoPCaC18.0 | lyso.PC.a.C18.0 | 524,4 | 184 | 26,675 | 26,2 | (M+H)+ | 1 |  |  |  |  |  |  |  |
| lysoPCaC18.1 | lyso.PC.a.C18.1 | 522,4 | 184 | 18,275 | 18,05 | (M+H)+ | 1 |  |  |  |  |  |  |  |
| lysoPCaC18.2 | lyso.PC.a.C18.2 | 520,3 | 184 | 32,6 | 32,15 | (M+H)+ | 1 |  |  |  |  |  |  |  |
| lysoPCaC20.3 | lyso.PC.a.C20.3 | 546,4 | 184 | 1,96 | 2,03 | (M+H)+ | 1 |  |  |  |  |  |  |  |
| lysoPCaC20.4 | lyso.PC.a.C20.4 | 544,3 | 184 | 6,5375 | 6,48 | (M+H)+ | 1 |  |  |  |  |  |  |  |
| lysoPCaC24.0 | lyso.PC.a.C24.0 | 608,5 | 184 | 0,76625 | 0,8805 | (M+H)+ | 1 |  |  |  |  |  |  |  |
| lysoPCaC26.0 | lyso.PC.a.C26.0 | 636,5 | 184 | 1,815 | 2,22 | (M+H)+ | 1 |  |  |  |  |  |  |  |
| lysoPCaC26.1 | lyso.PC.a.C26.1 | 634,5 | 184 | 3,4275 | 3,71 | (M+H)+ | 1 |  |  |  |  |  |  |  |
| lysoPCaC28.0 | lyso.PC.a.C28.0 | 664,5 | 184 | 1,3125 | 1,535 | (M+H)+ | 1 |  |  |  |  |  |  |  |
| lysoPCaC28.1 | lyso.PC.a.C28.1 | 662,5 | 184 | 1,52 | 1,785 | (M+H)+ | 1 |  |  |  |  |  |  |  |
| lysoPCaC6.0 | lyso.PC.a.C6.0 | 356,2 | 184 | 0,05025 | 0,0455 | (M+H)+ | 1 |  |  |  |  |  |  |  |
| PCaaC24.0 | PC.aa.C24.0 | 622,4 | 184 | 0,4935 | 0,596 | (M+H)+ | 1 |  |  |  |  |  |  |  |
| PCaaC26.0 | PC.aa.C26.0 | 650,5 | 184 | 2,735 | 3,33 | (M+H)+ | 1 |  |  |  |  |  |  |  |
| PCaaC28.1 | PC.aa.C28.1 | 676,5 | 184 | 2,6525 | 2,775 | (M+H)+ | 1 |  |  |  |  |  |  |  |
| PCaaC30.0 | PC.aa.C30.0 | 706,5 | 184 | 3,49 | 3,535 | (M+H)+ | 1 |  |  |  |  |  |  |  |
| PCaaC30.2 | PC.aa.C30.2 | 702,5 | 184 | 0,51625 | 0,548 | (M+H)+ | 1 |  |  |  |  |  |  |  |
| PCaaC32.0 | PC.aa.C32.0 | 734,6 | 184 | 11 | 11,05 | (M+H)+ | 1 |  |  |  |  |  |  |  |
| PCaaC32.1 | PC.aa.C32.1 | 732,6 | 184 | 13,3 | 13,75 | (M+H)+ | 1 |  |  |  |  |  |  |  |
| PCaaC32.2 | PC.aa.C32.2 | 730,5 | 184 | 2,8125 | 2,87 | (M+H)+ | 1 |  |  |  |  |  |  |  |
| PCaaC32.3 | PC.aa.C32.3 | 728,5 | 184 | 0,5415 | 0,525 | (M+H)+ | 1 |  |  |  |  |  |  |  |
| PCaaC34.1 | PC.aa.C34.1 | 760,6 | 184 | 167,5 | 167,5 | (M+H)+ | 1 |  |  |  |  |  |  |  |
| PCaaC34.2 | PC.aa.C34.2 | 758,6 | 184 | 278,75 | 282 | (M+H)+ | 1 |  |  |  |  |  |  |  |
| PCaaC34.3 | PC.aa.C34.3 | 756,6 | 184 | 14,65 | 13,6 | (M+H)+ | 1 |  |  |  |  |  |  |  |
| PCaaC34.4 | PC.aa.C34.4 | 754,5 | 184 | 1,675 | 1,65 | (M+H)+ | 1 |  |  |  |  |  |  |  |
| PCaaC36.0 | PC.aa.C36.0 | 790,6 | 184 | 2,305 | 2,095 | (M+H)+ | 1 |  |  |  |  |  |  |  |
| PCaaC36.1 | PC.aa.C36.1 | 788,6 | 184 | 35,4 | 34,15 | (M+H)+ | 1 |  |  |  |  |  |  |  |
| PCaaC36.2 | PC.aa.C36.2 | 786,6 | 184 | 170,5 | 173 | (M+H)+ | 1 |  |  |  |  |  |  |  |
| PCaaC36.3 | PC.aa.C36.3 | 784,6 | 184 | 97,8 | 98,9 | (M+H)+ | 1 |  |  |  |  |  |  |  |
| PCaaC36.4 | PC.aa.C36.4 | 782,6 | 184 | 125 | 127 | (M+H)+ | 1 |  |  |  |  |  |  |  |
| PCaaC36.5 | PC.aa.C36.5 | 780,6 | 184 | 15,975 | 15,75 | (M+H)+ | 1 |  |  |  |  |  |  |  |
| PCaaC36.6 | PC.aa.C36.6 | 778,5 | 184 | 0,9525 | 0,848 | (M+H)+ | 1 |  |  |  |  |  |  |  |
| PCaaC38.0 | PC.aa.C38.0 | 818,7 | 184 | 1,8325 | 1,81 | (M+H)+ | 1 |  |  |  |  |  |  |  |
| PCaaC38.1 | PC.aa.C38.1 | 816,6 | 184 | 1,17 | 0,9875 | (M+H)+ | 1 |  |  |  |  |  |  |  |
| PCaaC38.3 | PC.aa.C38.3 | 812,6 | 184 | 29,275 | 28,55 | (M+H)+ | 1 |  |  |  |  |  |  |  |
| PCaaC38.4 | PC.aa.C38.4 | 810,6 | 184 | 69,225 | 72,3 | (M+H)+ | 1 |  |  |  |  |  |  |  |
| PCaaC38.5 | PC.aa.C38.5 | 808,6 | 184 | 34,775 | 35,8 | (M+H)+ | 1 |  |  |  |  |  |  |  |
| PCaaC38.6 | PC.aa.C38.6 | 806,6 | 184 | 47,4 | 49,6 | (M+H)+ | 1 |  |  |  |  |  |  |  |
| PCaaC40.1 | PC.aa.C40.1 | 844,7 | 184 | 0,457 | 0,425 | (M+H)+ | 1 |  |  |  |  |  |  |  |
| PCaaC40.2 | PC.aa.C40.2 | 842,7 | 184 | 0,51675 | 0,436 | (M+H)+ | 1 |  |  |  |  |  |  |  |
| PCaaC40.3 | PC.aa.C40.3 | 840,6 | 184 | 0,72825 | 0,646 | (M+H)+ | 1 |  |  |  |  |  |  |  |
| PCaaC40.4 | PC.aa.C40.4 | 838,6 | 184 | 2,4325 | 2,51 | (M+H)+ | 1 |  |  |  |  |  |  |  |
| PCaaC40.5 | PC.aa.C40.5 | 836,6 | 184 | 6,835 | 7,19 | (M+H)+ | 1 |  |  |  |  |  |  |  |
| PCaaC40.6 | PC.aa.C40.6 | 834,6 | 184 | 15,6 | 16,6 | (M+H)+ | 1 |  |  |  |  |  |  |  |
| PCaaC42.0 | PC.aa.C42.0 | 874,7 | 184 | 0,4535 | 0,4445 | (M+H)+ | 1 |  |  |  |  |  |  |  |
| PCaaC42.1 | PC.aa.C42.1 | 872,7 | 184 | 0,2585 | 0,245 | (M+H)+ | 1 |  |  |  |  |  |  |  |
| PCaaC42.2 | PC.aa.C42.2 | 870,7 | 184 | 0,2725 | 0,233 | (M+H)+ | 1 |  |  |  |  |  |  |  |
| PCaaC42.4 | PC.aa.C42.4 | 866,7 | 184 | 0,248 | 0,222 | (M+H)+ | 1 |  |  |  |  |  |  |  |
| PCaaC42.5 | PC.aa.C42.5 | 864,6 | 184 | 0,3165 | 0,3175 | (M+H)+ | 1 |  |  |  |  |  |  |  |
| PCaaC42.6 | PC.aa.C42.6 | 862,6 | 184 | 0,55475 | 0,58 | (M+H)+ | 1 |  |  |  |  |  |  |  |
| PCaeC30.0 | PC.ae.C30.0 | 692,6 | 184 | 0,432 | 0,4415 | (M+H)+ | 1 |  |  |  |  |  |  |  |
| PCaeC30.1 | PC.ae.C30.1 | 690,5 | 184 | 0,691 | 0,882 | (M+H)+ | 1 |  |  |  |  |  |  |  |
| PCaeC30.2 | PC.ae.C30.2 | 688,5 | 184 | 0,249 | 0,2505 | (M+H)+ | 1 |  |  |  |  |  |  |  |
| PCaeC32.1 | PC.ae.C32.1 | 718,6 | 184 | 2,2625 | 2,28 | (M+H)+ | 1 |  |  |  |  |  |  |  |
| PCaeC32.2 | PC.ae.C32.2 | 716,6 | 184 | 0,798 | 0,8295 | (M+H)+ | 1 |  |  |  |  |  |  |  |
| PCaeC34.0 | PC.ae.C34.0 | 748,6 | 184 | 1,3025 | 1,275 | (M+H)+ | 1 |  |  |  |  |  |  |  |
| PCaeC34.1 | PC.ae.C34.1 | 746,6 | 184 | 7,3125 | 7,285 | (M+H)+ | 1 |  |  |  |  |  |  |  |
| PCaeC34.2 | PC.ae.C34.2 | 744,6 | 184 | 8,33 | 8,28 | (M+H)+ | 1 |  |  |  |  |  |  |  |
| PCaeC34.3 | PC.ae.C34.3 | 742,6 | 184 | 5,845 | 6,035 | (M+H)+ | 1 |  |  |  |  |  |  |  |
| PCaeC36.0 | PC.ae.C36.0 | 776,7 | 184 | 0,765 | 0,7355 | (M+H)+ | 1 |  |  |  |  |  |  |  |
| PCaeC36.1 | PC.ae.C36.1 | 774,6 | 184 | 7,6675 | 6,57 | (M+H)+ | 1 |  |  |  |  |  |  |  |
| PCaeC36.2 | PC.ae.C36.2 | 772,6 | 184 | 11,525 | 10,75 | (M+H)+ | 1 |  |  |  |  |  |  |  |
| PCaeC36.3 | PC.ae.C36.3 | 770,6 | 184 | 5,5775 | 5,515 | (M+H)+ | 1 |  |  |  |  |  |  |  |
| PCaeC36.4 | PC.ae.C36.4 | 768,6 | 184 | 11,05 | 11,15 | (M+H)+ | 1 |  |  |  |  |  |  |  |
| PCaeC36.5 | PC.ae.C36.5 | 766,6 | 184 | 7,2275 | 7,405 | (M+H)+ | 1 |  |  |  |  |  |  |  |
| PCaeC38.0 | PC.ae.C38.0 | 804,7 | 184 | 2,195 | 2,045 | (M+H)+ | 1 |  |  |  |  |  |  |  |
| PCaeC38.1 | PC.ae.C38.1 | 802,7 | 184 | 1,8875 | 1,41 | (M+H)+ | 1 |  |  |  |  |  |  |  |
| PCaeC38.2 | PC.ae.C38.2 | 800,7 | 184 | 3,125 | 2,535 | (M+H)+ | 1 |  |  |  |  |  |  |  |
| PCaeC38.3 | PC.ae.C38.3 | 798,6 | 184 | 5,1625 | 4,68 | (M+H)+ | 1 |  |  |  |  |  |  |  |
| PCaeC38.4 | PC.ae.C38.4 | 796,6 | 184 | 9,3975 | 9,28 | (M+H)+ | 1 |  |  |  |  |  |  |  |
| PCaeC38.5 | PC.ae.C38.5 | 794,6 | 184 | 10,525 | 10,55 | (M+H)+ | 1 |  |  |  |  |  |  |  |
| PCaeC38.6 | PC.ae.C38.6 | 792,6 | 184 | 4,71 | 4,805 | (M+H)+ | 1 |  |  |  |  |  |  |  |
| PCaeC40.0 | PC.ae.C40.0 | 832,7 | 184 | 7,225 | 7,365 | (M+H)+ | 1 |  |  |  |  |  |  |  |
| PCaeC40.1 | PC.ae.C40.1 | 830,7 | 184 | 1,555 | 1,55 | (M+H)+ | 1 |  |  |  |  |  |  |  |
| PCaeC40.2 | PC.ae.C40.2 | 828,7 | 184 | 1,7025 | 1,6 | (M+H)+ | 1 |  |  |  |  |  |  |  |
| PCaeC40.3 | PC.ae.C40.3 | 826,7 | 184 | 1,65 | 1,45 | (M+H)+ | 1 |  |  |  |  |  |  |  |
| PCaeC40.4 | PC.ae.C40.4 | 824,7 | 184 | 2,0575 | 1,955 | (M+H)+ | 1 |  |  |  |  |  |  |  |
| PCaeC40.5 | PC.ae.C40.5 | 822,6 | 184 | 3,285 | 3,24 | (M+H)+ | 1 |  |  |  |  |  |  |  |
| PCaeC40.6 | PC.ae.C40.6 | 820,6 | 184 | 2,885 | 2,93 | (M+H)+ | 1 |  |  |  |  |  |  |  |
| PCaeC42.0 | PC.ae.C42.0 | 860,7 | 184 | 0,52825 | 0,533 | (M+H)+ | 1 |  |  |  |  |  |  |  |
| PCaeC42.1 | PC.ae.C42.1 | 858,7 | 184 | 0,53275 | 0,5395 | (M+H)+ | 1 |  |  |  |  |  |  |  |
| PCaeC42.2 | PC.ae.C42.2 | 856,7 | 184 | 0,54675 | 0,5235 | (M+H)+ | 1 |  |  |  |  |  |  |  |
| PCaeC42.3 | PC.ae.C42.3 | 854,7 | 184 | 1,065 | 0,9655 | (M+H)+ | 1 |  |  |  |  |  |  |  |
| PCaeC42.4 | PC.ae.C42.4 | 852,7 | 184 | 0,83025 | 0,7745 | (M+H)+ | 1 |  |  |  |  |  |  |  |
| PCaeC42.5 | PC.ae.C42.5 | 850,7 | 184 | 1,765 | 1,76 | (M+H)+ | 1 |  |  |  |  |  |  |  |
| PCaeC44.3 | PC.ae.C44.3 | 882,7 | 184 | 0,28225 | 0,219 | (M+H)+ | 1 |  |  |  |  |  |  |  |
| PCaeC44.4 | PC.ae.C44.4 | 880,7 | 184 | 0,4375 | 0,412 | (M+H)+ | 1 |  |  |  |  |  |  |  |
| PCaeC44.5 | PC.ae.C44.5 | 878,7 | 184 | 1,4725 | 1,51 | (M+H)+ | 1 |  |  |  |  |  |  |  |
| PCaeC44.6 | PC.ae.C44.6 | 876,7 | 184 | 0,85425 | 0,8785 | (M+H)+ | 1 |  |  |  |  |  |  |  |
| SM.OH.C14.1 | SM.C18.1.OH.C14.1 | 689,5 | 184 | 4,4825 | 4,26 | (M+H)+ | 1 |  |  |  |  |  |  |  |
| SM.OH.C16.1 | SM.C18.1.OH.C16.1 | 717,6 | 184 | 2,065 | 2,075 | (M+H)+ | 1 |  |  |  |  |  |  |  |
| SM.OH.C22.1 | SM.C18.1.OH.C22.1 | 801,6 | 184 | 8,58 | 8,365 | (M+H)+ | 1 |  |  |  |  |  |  |  |
| SM.OH.C22.2 | SM.C18.1.OH.C22.2 | 799,6 | 184 | 6,26 | 6,14 | (M+H)+ | 1 |  |  |  |  |  |  |  |
| SM.OH.C24.1 | SM.C18.1.OH.C24.1 | 829,7 | 184 | 0,9405 | 0,9585 | (M+H)+ | 1 |  |  |  |  |  |  |  |
| SMC16.0 | SM.C18.1.C16.0 | 703,6 | 184 | 76,4 | 75 | (M+H)+ | 1 |  |  |  |  |  |  |  |
| SMC16.1 | SM.C18.1.C16.1 | 701,6 | 184 | 10,525 | 10,55 | (M+H)+ | 1 |  |  |  |  |  |  |  |
| SMC18.0 | SM.C18.1.C18.0 | 731,6 | 184 | 14,575 | 14,35 | (M+H)+ | 1 |  |  |  |  |  |  |  |
| SMC18.1 | SM.C18.1.C18.1 | 729,6 | 184 | 7,1225 | 7,025 | (M+H)+ | 1 |  |  |  |  |  |  |  |
| SMC20.2 | SM.C18.1.C20.2 | 755,6 | 184 | 0,322 | 0,282 | (M+H)+ | 1 |  |  |  |  |  |  |  |
| SMC22.3 | SM.C18.1.C22.3 | 781,6 | 184 | 1,7625 | 1,755 | (M+H)+ | 1 |  |  |  |  |  |  |  |
| SMC24.0 | SM.C18.1.C24.0 | 815,7 | 184 | 14,8 | 14,3 | (M+H)+ | 1 |  |  |  |  |  |  |  |
| SMC24.1 | SM.C18.1.C24.1 | 813,7 | 184 | 33 | 32,15 | (M+H)+ | 1 |  |  |  |  |  |  |  |
| SMC26.0 | SM.C18.1.C26.0 | 843,7 | 184 | 0,07225 | 0,0815 | (M+H)+ | 1 |  |  |  |  |  |  |  |
| SMC26.1 | SM.C18.1.C26.1 | 841,7 | 184 | 0,28175 | 0,2855 | (M+H)+ | 1 |  |  |  |  |  |  |  |
| Sum of Hexoses | Sum of Hexoses | 179 | 89 | 22237,5 | 22592 | (M+H)+ | 1 |  |  |  |  |  |  |  |
| ID metabolite identity metabolomics laboratory. Sofia ID metabolite identity Generation R Study group. Q1/Q3 Quadrupole 1 and 3, CP1/CP2 Calibrators 1 and 2, MSI ID Metabolomics Standards Initiative identification.  This table is adapted from: Voerman, E., Jaddoe, V. W. V., Uhl, O., & Shokry, E. (2020). A population based resource for intergenerational metabolomics analysis in pregnant women and their children : the Generation R Study. *Metabolomics*, 1 – 26. Voerman et al. 2020 describes the acquisition, processing and structure of the metabolomics data in the Generation R study cohort. | | | | | | | |  |  |  |  |  |  | 1 |

**Supplementary table S5.** Cord blood metabolite concentrations

| **Neonatal metabolite or metabolite group** | **Median (95% range), μmol/L** |
| --- | --- |
| **Amino acids (AA)** | **3587.85 (2391.97 - 4825.57)** |
| **BCAA** | **445.03 (277.65 - 649.81)** |
| **AAA** | **234.62 (152.64 - 328.72)** |
| **Essential AA** | **1385.55 (931.84 - 1911.35)** |
| **Non-essential AA** | **2185.38 (1438.56 - 3104.90)** |
| Ala | 548.96 (330.27 - 1007.26) |
| Arg | 70.41 (28.76 - 129.54) |
| Asn | 52.07 (32.07 - 85.81) |
| Asp | 35.11 (20.33 - 72.40) |
| Cit | 13.67 (7.54 - 21.96) |
| Gln | 403.16 (229.98 - 759.63) |
| Glu | 176.06 (97.58 - 357.09) |
| Gly | 297.04 (198.69 - 435.42) |
| His | 123.01 (76.81 - 196.05) |
| Ile | 68.30 (39.61 - 105.13) |
| Leu | 130.59 (75.40 - 199.24) |
| Lys | 349.87 (217.70 - 571.07) |
| Met | 30.53 (19.33 - 46.07) |
| Orn | 110.24 (59.11 - 182.26) |
| Phe | 95.83 (61.23 - 139.16) |
| Pro | 167.43 (112.72 - 297.72) |
| Trp | 69.04 (40.66 - 112.04) |
| Ser | 141.05 (90.13 - 319.61) |
| Thr | 253.58 (151.19 - 460.44) |
| Tyr | 67.75 (39.34 - 101.01) |
| Val | 246.11 (156.19 - 343.31) |
| Cys | 17.51 (7.66 - 40.87) |
| **Non-esterified fatty acids (NEFA)** | **187.62 (78.25 - 393.93)** |
| **Saturated NEFA** | **87.80 (35.35 - 184.77)** |
| **Mono-unsaturated NEFA** | **55.44 (20.26 - 132.97)** |
| **Poly-unsaturated NEFA** | **43.59 (17.66 - 90.38)** |
| NEFA_14_0 | 7.21 (2.36 - 17.51) |
| NEFA_14_1 | 1.57 (0.36 - 4.83) |
| NEFA_15_0 | 1.05 (0.40 - 2.49) |
| NEFA_16_0 | 65.77 (25.51 - 139.57) |
| NEFA_16_1 | 9.74 (2.93 - 28.40) |
| NEFA_16_2 | 0.46 (0.15 - 1.15) |
| NEFA_17_0 | 1.11 (0.44 - 2.23) |
| NEFA_17_1 | 0.52 (0.10 - 1.35) |
| NEFA_17_2 | 0.04 (0.00 - 0.09) |
| NEFA_18_0 | 12.84 (2.13 - 29.98) |
| NEFA_18_1 | 42.06 (16.02 - 95.19) |
| NEFA_18_2 | 23.33 (7.75 - 56.89) |
| NEFA_18_3 | 2.09 (0.29 - 5.32) |
| NEFA_19_1 | 0.15 (0.05 - 0.33) |
| NEFA_20_1 | 0.39 (0.12 - 0.87) |
| NEFA_20_2 | 0.54 (0.21 - 1.15) |
| NEFA_20_3 | 1.85 (0.79 - 4.21) |
| NEFA_20_4 | 7.77 (3.95 - 15.00) |
| NEFA_20_5 | 0.27 (0.09 - 0.72) |
| NEFA_22_3 | 0.14 (0.08 - 0.28) |
| NEFA_22_4 | 0.62 (0.33 - 1.18) |
| NEFA_22_5 | 0.76 (0.34 - 1.58) |
| NEFA_22_6 | 4.18 (1.86 - 9.94) |
| NEFA_24_0 | 0.18 (0.11 - 0.30) |
| NEFA_24_1 | 0.17 (0.08 - 0.30) |
| NEFA_24_2 | 0.10 (0.05 - 0.20) |
| NEFA_24_4 | 0.12 (0.07 - 0.24) |
| NEFA_24_5 | 0.11 (0.06 - 0.22) |
| NEFA_26_0 | 0.14 (0.07 - 0.29) |
| NEFA_26_1 | 0.09 (0.06 - 0.16) |
| NEFA_26_2 | 0.06 (0.04 - 0.10) |
| **Acyl-lysosphophatidylcholines (LysoPCa)** | **137.42 (76.33 - 219.09)** |
| **Saturated LysoPCa** | **88.69 (53.00 - 140.46)** |
| **Mono-unsaturated LysoPCa** | **16.95 (8.21 - 30.08)** |
| **Poly-unsaturated LysoPCa** | **30.26 (14.75 - 55.17)** |
| Lyso.PC.a.C14.0 | 3.01 (1.32 - 5.57) |
| Lyso.PC.a.C16.0 | 70.18 (40.81 - 111.66) |
| Lyso.PC.a.C16.1 | 4.47 (1.96 - 9.09) |
| Lyso.PC.a.C18.0 | 14.64 (9.19 - 24.41) |
| Lyso.PC.a.C18.1 | 12.75 (6.18 - 22.26) |
| Lyso.PC.a.C18.2 | 11.52 (4.98 - 21.50) |
| Lyso.PC.a.C18.3 | 0.27 (0.07 - 0.60) |
| Lyso.PC.a.C20.3 | 3.64 (1.40 - 6.98) |
| Lyso.PC.a.C20.4 | 12.29 (5.78 - 22.44) |
| Lyso.PC.a.C20.5 | 0.28 (0.05 - 0.72) |
| Lyso.PC.a.C22.6 | 2.39 (1.27 - 4.84) |
| **Alkyl-lysophosphatidylcholines (LysoPCe)** | **1.70 (0.86 - 2.93)** |
| **Saturated LysoPCe** | **1.40 (0.69 - 2.55)** |
| **Mono-unsaturated LysoPCe** | **0.28 (0.13 - 0.51)** |
| Lyso.PC.e.C16.0 | 0.52 (0.24 - 0.98) |
| Lyso.PC.e.C18.0 | 0.87 (0.35 - 1.62) |
| Lyso.PC.e.C18.1 | 0.28 (0.13 - 0.51) |
| **Diacyl-phosphatidylcholines (PCaa)** | **718.98 (425.38 - 1245.83** |
| **Saturated PCaa** | **17.05 (9.74 - 31.14)** |
| **Mono-unsaturated PCaa** | **121.32 (68.08 - 208.77)** |
| **Poly-unsaturated PCaa** | **575.20 (336.79 - 983.93)** |
| PC.aa.C30.0 | 2.70 (1.21 - 5.27) |
| PC.aa.C30.3 | 0.12 (0.05 - 0.27) |
| PC.aa.C32.0 | 10.93 (5.87 - 20.30) |
| PC.aa.C32.1 | 8.99 (4.41 - 21.08) |
| PC.aa.C32.2 | 0.67 (0.07 - 2.07) |
| PC.aa.C32.3 | 0.29 (0.10 - 0.61) |
| PC.aa.C34.1 | 93.71 (50.86 - 160.47) |
| PC.aa.C34.2 | 61.23 (31.34 - 121.22) |
| PC.aa.C34.3 | 2.39 (1.14 - 5.26) |
| PC.aa.C34.4 | 0.38 (0.19 - 0.81) |
| PC.aa.C34.5 | 0.05 (0.01 - 0.11) |
| PC.aa.C36.0 | 1.00 (0.41 - 2.10) |
| PC.aa.C36.1 | 19.04 (11.15 - 33.04) |
| PC.aa.C36.2 | 37.30 (19.83 - 69.82) |
| PC.aa.C36.3 | 61.76 (32.45 - 108.77) |
| PC.aa.C36.4 | 130.91 (67.80 - 225.30) |
| PC.aa.C36.5 | 4.62 (2.21 - 10.25) |
| PC.aa.C36.6 | 0.23 (0.05 - 0.50) |
| PC.aa.C38.0 | 1.35 (0.55 - 3.03) |
| PC.aa.C38.2 | 2.89 (1.08 - 6.19) |
| PC.aa.C38.3 | 42.16 (18.57 - 76.76) |
| PC.aa.C38.4 | 96.74 (51.55 - 175.12) |
| PC.aa.C38.5 | 21.17 (10.86 - 38.58) |
| PC.aa.C38.6 | 66.43 (35.10 - 130.23) |
| PC.aa.C40.0 | 0.51 (0.20 - 0.95) |
| PC.aa.C40.1 | 0.21 (0.03 - 0.64) |
| PC.aa.C40.2 | 0.14 (0.03 - 0.30) |
| PC.aa.C40.3 | 0.40 (0.13 - 0.75) |
| PC.aa.C40.4 | 2.76 (1.41 - 5.50) |
| PC.aa.C40.5 | 6.84 (3.14 - 13.45) |
| PC.aa.C40.6 | 29.16 (13.70 - 59.79) |
| PC.aa.C42.0 | 0.56 (0.27 - 1.20) |
| PC.aa.C42.5 | 0.35 (0.12 - 0.64) |
| PC.aa.C43.6 | 1.74 (0.91 - 3.57) |
| PC.aa.C44.12 | 0.27 (0.14 - 0.60) |
| **Acyl-alkyl-phosphatidylcholines (PCae)** | **68.41 (42.78 - 118.49)** |
| **Saturated PCae** | **11.45 (6.54 - 19.64)** |
| **Mono-unsaturated PCae** | **8.16 (4.74 - 14.06)** |
| **Poly-unsaturated PCae** | **49.69 (30.36 - 85.28)** |
| PC.ae.C30.0 | 0.22 (0.06 - 0.52) |
| PC.ae.C32.0 | 2.22 (1.08 - 4.37) |
| PC.ae.C32.1 | 2.02 (0.94 - 3.87) |
| PC.ae.C32.2 | 0.40 (0.17 - 0.91) |
| PC.ae.C34.0 | 0.80 (0.32 - 1.86) |
| PC.ae.C34.1 | 3.49 (1.83 - 6.50) |
| PC.ae.C34.2 | 2.22 (1.19 - 4.30) |
| PC.ae.C34.3 | 1.07 (0.51 - 2.31) |
| PC.ae.C34.4 | 0.07 (0.03 - 0.15) |
| PC.ae.C36.0 | 0.52 (0.27 - 1.00) |
| PC.ae.C36.1 | 1.74 (0.92 - 3.31) |
| PC.ae.C36.2 | 1.93 (1.04 - 3.76) |
| PC.ae.C36.3 | 1.81 (0.91 - 3.48) |
| PC.ae.C36.4 | 7.99 (4.54 - 14.23) |
| PC.ae.C36.5 | 6.14 (3.10 - 12.71) |
| PC.ae.C38.0 | 0.95 (0.43 - 1.77) |
| PC.ae.C38.2 | 0.49 (0.14 - 1.05) |
| PC.ae.C38.3 | 1.66 (0.83 - 2.92) |
| PC.ae.C38.4 | 6.70 (3.77 - 11.96) |
| PC.ae.C38.5 | 7.01 (3.96 - 12.85) |
| PC.ae.C38.6 | 3.26 (1.95 - 6.22) |
| PC.ae.C40.0 | 6.56 (3.05 - 12.28) |
| PC.ae.C40.1 | 0.67 (0.15 - 1.34) |
| PC.ae.C40.2 | 0.40 (0.04 - 1.24) |
| PC.ae.C40.3 | 0.44 (0.11 - 1.06) |
| PC.ae.C40.4 | 1.79 (0.80 - 3.31) |
| PC.ae.C40.5 | 1.41 (0.76 - 2.57) |
| PC.ae.C40.6 | 2.07 (1.09 - 4.11) |
| PC.ae.C42.1 | 0.26 (0.09 - 0.48) |
| PC.ae.C42.3 | 0.28 (0.06 - 0.68) |
| PC.ae.C42.4 | 0.53 (0.14 - 1.12) |
| PC.ae.C42.5 | 1.01 (0.47 - 2.52) |
| PC.ae.C42.6 | 0.85 (0.37 - 1.65) |
| **Sphingomyelines (SM)** | **204.68 (120.14 - 359.03)** |
| **Mono-unsaturated SM** | **100.36 (61.45 - 166.63)** |
| **Poly-unsaturated SM** | **106.27 (57.52 - 192.46)** |
| SM.a.C30.1 | 0.13 (0.04 - 0.29) |
| SM.a.C32.1 | 2.91 (1.50 - 5.26) |
| SM.a.C32.2 | 0.43 (0.23 - 0.82) |
| SM.a.C33.1 | 2.14 (1.05 - 3.80) |
| SM.a.C34.1 | 50.49 (28.97 - 92.31) |
| SM.a.C34.2 | 10.42 (5.49 - 19.91) |
| SM.a.C35.1 | 1.60 (0.86 - 3.30) |
| SM.a.C36.1 | 18.50 (9.78 - 30.95) |
| SM.a.C36.2 | 12.02 (5.66 - 23.00) |
| SM.a.C36.3 | 0.32 (0.12 - 0.66) |
| SM.a.C37.1 | 0.95 (0.40 - 1.91) |
| SM.a.C38.2 | 5.01 (2.42 - 10.98) |
| SM.a.C38.3 | 0.16 (0.06 - 0.30) |
| SM.a.C39.1 | 1.27 (0.49 - 2.94) |
| SM.a.C39.2 | 0.42 (0.08 - 0.82) |
| SM.a.C40.2 | 10.57 (4.52 - 25.18) |
| SM.a.C40.5 | 0.23 (0.10 - 0.50) |
| SM.a.C41.1 | 3.67 (1.63 - 7.04) |
| SM.a.C41.2 | 3.32 (1.40 - 6.88) |
| SM.a.C42.1 | 17.18 (9.16 - 29.66) |
| SM.a.C42.2 | 32.22 (17.47 - 63.28) |
| SM.a.C42.3 | 17.58 (6.94 - 35.50) |
| SM.a.C42.4 | 6.16 (2.98 - 11.99) |
| SM.a.C42.6 | 2.72 (1.26 - 5.41) |
| SM.a.C43.1 | 0.89 (0.37 - 1.89) |
| SM.a.C43.2 | 1.19 (0.54 - 2.25) |
| SM.a.C44.6 | 1.42 (0.53 - 2.65) |
| SM.e.C36.2 | 0.23 (0.10 - 0.46) |
| SM.e.C38.3 | 0.06 (0.01 - 0.15) |
| SM.e.C40.5 | 0.21 (0.09 - 0.43) |
| **Free Carn** | **15.32 (9.41 - 26.31)** |
| **Acyl-carnitine (Carn.a)** | **5.50 (3.06 - 10.33)** |
| **Small-chain Carn.a** | **4.34 (2.28 - 8.83)** |
| **Medium-chain Carn.a** | **0.46 (0.25 - 0.82)** |
| **Large-chain Carn.a** | **0.77 (0.42 - 1.28)** |
| Carn | 15.32 (9.41 - 26.31) |
| Carn.a.C10.0 | 0.08 (0.04 - 0.21) |
| Carn.a.C10.1 | 0.07 (0.04 - 0.12) |
| Carn.a.C12.0 | 0.08 (0.04 - 0.17) |
| Carn.a.C14.1 | 0.04 (0.02 - 0.11) |
| Carn.a.C14.2 | 0.03 (0.01 - 0.06) |
| Carn.a.C15.0 | 0.04 (0.02 - 0.06) |
| Carn.a.C16.0 | 0.15 (0.08 - 0.29) |
| Carn.a.C16.0.Oxo | 0.02 (0.01 - 0.03) |
| Carn.a.C16.1 | 0.10 (0.05 - 0.20) |
| Carn.a.C16.2 | 0.03 (0.01 - 0.05) |
| Carn.a.C18.0 | 0.09 (0.05 - 0.16) |
| Carn.a.C18.1 | 0.08 (0.04 - 0.16) |
| Carn.a.C18.2 | 0.06 (0.03 - 0.11) |
| Carn.a.C18.2.OH | 0.02 (0.01 - 0.04) |
| Carn.a.C2.0 | 3.65 (1.83 - 7.70) |
| Carn.a.C20.0 | 0.03 (0.02 - 0.06) |
| Carn.a.C20.1 | 0.00 (0.00 - 0.00) |
| Carn.a.C20.3 | 0.05 (0.02 - 0.09) |
| Carn.a.C20.4 | 0.00 (0.00 - 0.01) |
| Carn.a.C3.0 | 0.29 (0.14 - 0.59) |
| Carn.a.C3.0.DC | 0.10 (0.04 - 0.36) |
| Carn.a.C4.0 | 0.13 (0.07 - 0.24) |
| Carn.a.C5.0 | 0.13 (0.06 - 0.31) |
| Carn.a.C6.0 | 0.05 (0.02 - 0.10) |
| Carn.a.C6.0.OH | 0.04 (0.02 - 0.08) |
| Carn.a.C8.0 | 0.05 (0.02 - 0.13) |
| Carn.a.C8.1 | 0.05 (0.02 - 0.11) |
| Carn.a.C9.0 | 0.02 (0.01 - 0.04) |
| Values represent medians (95% range) of neonatal metabolite concentrations in cordblood (μmol/L). | |

**Supplementary table S6.** Comparison of general characteristics between mother-child pairs with bisphenol measurement and mother-child pairs without bisphenol measurement

|  | **Participants**  n = 225 | **Non-participants ^a^**  n = 688 | **P-value** |
| --- | --- | --- | --- |
| **Maternal characteristics** |  |  |  |
| Age at enrolment, mean (±SD), years | 31.9 (3.7) | 31.4 (4.3) | 0.160 |
| Parity, *n* (%) |  |  | 0.031* |
| Nullipara | 150 (66.7) | 403 (58.6) |  |
| Multipara | 75 (33.3) | 285 (41.1) |  |
| Ethnicity, *n* (%) |  |  | NA |
| Dutch | 225 (100.0) | 668 (100.0) |  |
| Other | 0 (0.0) | 0 (0.0) |  |
| Education*, n* (%) |  |  | 0.503 |
| Primary | 3 (1.3) | 17 (2.5) |  |
| Secondary | 76 (33.8) | 243 (35.3) |  |
| Higher | 145 (64.4) | 422 (61.3) |  |
| Pre-pregnancy BMI, median (95% range), kg/m^2^ | 22.6 (18.6-35.6) | 22.3 (18.4 – 33.0) | 0.434 |
| Smoking, *n* (%) |  |  | 0.038* |
| Never smoked during pregnancy | 161 (71.6) | 471 (68.5) |  |
| Smoked until pregnancy was known | 14 (6.2) | 63 (9.2) |  |
| Continued smoking in pregnancy | 19 (8.4) | 101 (14.7) |  |
| Alcohol consumption, *n* (%) |  |  | 0.141 |
| Never alcohol in pregnancy | 53 (23.6) | 212 (30.8) |  |
| Alcohol until pregnancy was known | 38 (16.9) | 94 (13.7) |  |
| Alcohol continued in pregnancy | 103 (45.8) | 326 (47.4) |  |
|  |  |  |  |
| **Child characteristics** |  |  |  |
| Gestational age at birth in weeks, median (95% range) | 40.4 (37.3-42.3) | 40.3 (36.5 – 42.4) | 0.770 |
| Premature birth, *n* (%) | 3 (1.3) | 23 (3.3) | 0.116 |
| Sex, *n* (%) |  |  | 0.232 |
| Male | 129 (57.3) | 363 (52.8) |  |
| Female | 96 (42.7) | 325 (47.2) |  |
| Birthweight, mean (±SD), gram | 3538.8 (453.5) | 3546.2 (518.2) | 0.849 |
| SGA, *n* (%) | 22 (9.8) | 68 (9.9) |  |
| LGA, *n* (%) | 22 (9.8) | 74 (10.8) |  |
| Low birthweight, *n* (%) | 2 (0.9) | 18 (2.6) | 0.124 |
| Macrosomia, *n* (%) | 31 (13.8) | 123 (17.9) | 0.152 |
| Values represent mean (SD), median (95% range) or number of participants (valid %). *p-value<0.05. ^a^ Dutch mothers with singleton live-born children with cord blood sampling for metabolomics without maternal urinary bisphenol measurement in pregnancy.  Premature birth, birth at gestational age <37 weeks; SGA, small for gestational age, gestational age adjusted birthweight <10^th^ percentile; LGA, large for gestational age, gestational age adjusted birthweight >90^th^ percentil; low birthweight, birthweight <2500 kilogram; macrosomia, birthweight >4000 kilogram. | | | |

**Supplementary table S7.** Cord blood metabolite concentrations in mother-child pairs with bisphenol measurement and mother-child pairs without bisphenol measurement

|  | **Participants**  n = 225 | **Non-participants ^a^**  n = 688 | **P-value** |
| --- | --- | --- | --- |
| **Neonatal metabolite group** | **Median (95% range), μmol/L** | **Median (95% range), μmol/L** |  |
| Amino acids (AA) | 3587.85 (2391.97 - 4825.57) | 3910.18 (2671.29 – 5383.33) | 0.000* |
| BCAA | 445.03 (277.65 - 649.81) | 488.46 (334.86 – 722.11) | 0.000* |
| AAA | 234.62 (152.64 - 328.72) | 262.90 (176.16 – 375.91) | 0.000* |
| Essential AA | 1385.55 (931.84 - 1911.35) | 1482.02 (1047.01 – 2057.49) | 0.000* |
| Non-essential AA | 2185.38 (1438.56 - 3104.90) | 2430.99 (1568.09 – 3436.96) | 0.000* |
| Non-esterified fatty acids (NEFA) | 187.62 (78.25 - 393.93) | 190.86 (82.09 – 409.88) | 0.333 |
| Saturated NEFA | 87.80 (35.35 - 184.77) | 90.74 (36.98 – 189.49) | 0.280 |
| Mono-unsaturated NEFA | 55.44 (20.26 - 132.97) | 56.02 (21.27 – 134.36) | 0.562 |
| Poly-unsaturated NEFA | 43.59 (17.66 - 90.38) | 44.22 (19.79 – 93.55) | 0.247 |
| Acyl-lysosphophatidylcholines (Lyso.PC.a) | 137.42 (76.33 - 219.09) | 145.88 (87.57 – 230.48) | 0.005* |
| Saturated Lyso.PC.a | 88.69 (53.00 - 140.46) | 92.77 (56.04 – 152.13) | 0.026* |
| Mono-unsaturated Lyso.PC.a | 16.95 (8.21 - 30.08) | 17.96 (9.43 – 32.59) | 0.008* |
| Poly-unsaturated Lyso.PC.a | 30.26 (14.75 - 55.17) | 33.02 (17.79 – 57.31) | 0.000* |
| Alkyl-lysophosphatidylcholines (Lyso.PC.e) | 1.70 (0.86 - 2.93) | 1.65 (0.82 – 3.02) | 0.878 |
| Saturated Lyso.PC.e | 1.40 (0.69 - 2.55) | 1.37 (0.65 – 2.60) | 0.790 |
| Mono-unsaturated Lyso.PC.e | 0.28 (0.13 - 0.51) | 0.27 (0.10 – 0.50) | 0.825 |
| Diacyl-phosphatidylcholines (PC.aa) | 718.98 (425.38 - 1245.83 | 761.46 (466.00 – 1291.96) | 0.001* |
| Saturated PC.aa | 17.05 (9.74 - 31.14) | 18.33 (10.55 – 33.40) | 0.001* |
| Mono-unsaturated PC.aa | 121.32 (68.08 - 208.77) | 129.46 (74.56 – 249.30) | 0.004* |
| Poly-unsaturated PC.aa | 575.20 (336.79 - 983.93) | 613.59 (382.61 – 1030.93) | 0.002* |
| Acyl-alkyl-phosphatidylcholines (PC.ae) | 68.41 (42.78 - 118.49) | 74.73 (48.12 – 129.94) | 0.001* |
| Saturated PC.ae | 11.45 (6.54 - 19.64) | 12.21 (7.38 – 21.46) | 0.001* |
| Mono-unsaturated PC.ae | 8.16 (4.74 - 14.06) | 8.64 (5.12 – 16.18) | 0.006* |
| Poly-unsaturated PC.ae | 49.69 (30.36 - 85.28) | 53.89 (33.71 – 94.09) | 0.001* |
| Sphingomyelines (SM) | 204.68 (120.14 - 359.03) | 224.81 (139.67 – 384.75) | 0.000* |
| Mono-unsaturated SM | 100.36 (61.45 - 166.63) | 109.19 (68.86 – 184.53) | 0.000* |
| Poly-unsaturated SM | 106.27 (57.52 - 192.46) | 115.48 (69.55 – 208.45) | 0.000* |
| Free Carn | 15.32 (9.41 - 26.31) | 16.64 (9.45 – 28.02) | 0.000* |
| Acyl-carnitine (Carn.a) | 5.50 (3.06 - 10.33) | 5.94 (3.61 – 10.09) | 0.005* |
| Small-chain Carn.a | 4.34 (2.28 - 8.83) | 4.50 (2.53 – 8.43) | 0.111 |
| Medium-chain Carn.a | 0.46 (0.25 - 0.82) | 0.53 (0.30 – 0.89) | 0.000* |
| Large-chain Carn.a | 0.77 (0.42 - 1.28) | 0.88 (0.50 – 1.35) | 0.000* |
| Values represent medians (95% range) of neonatal metabolite concentrations in cordblood (μmol/L). *p-value<0.05. ^a^ Dutch mothers with singleton live-born children with cord blood sampling for metabolomics without maternal urinary bisphenol measurement in pregnancy. | | | |

**Supplementary table S8.** Comparison of general characteristics between mother-child pairs with metabolite measurement and mother-child pairs without metabolite measurement

|  | **Participants**  n = 225 | **Non-participants ^a^**  n = 507 | **P-value** |
| --- | --- | --- | --- |
| **Maternal characteristics** |  |  |  |
| Age at enrolment, mean (±SD), years | 31.9 (3.7) | 31.5 (4.0) | 0.320 |
| Parity, *n* (%) |  |  | 0.717 |
| Nullipara | 150 (66.7) | 331 (65.3) |  |
| Multipara | 75 (33.3) | 176 (34.7) |  |
| Ethnicity, *n* (%) |  |  | NA |
|  | 225 (100) | 507 (100) |  |
|  | 0 (0.0) | 0 (0.0) |  |
| Education, *n* (%) |  |  | 0.788 |
| Primary | 3 (1.3) | 10 (2.0) |  |
| Secondary | 76 (33.9) | 163 (32.5) |  |
| Higher | 145 (64.7) | 328 (65.5) |  |
| Pre-pregnancy BMI, median (95% range), kg/m^2^ | 22.6 (18.6 – 35.5) | 22.2 (18.4 – 34.6) | 0.120 |
| Smoking, *n* (%) |  |  | 0.322 |
| Never smoked during pregnancy | 161 (83.0) | 369 (77.8) |  |
| Smoked until pregnancy was known | 14 (7.2) | 47 (9.9) |  |
| Continued smoking in pregnancy | 19 (9.8) | 58 (12.2) |  |
| Alcohol consumption, *n* (%) |  |  | 0.141 |
| Never alcohol in pregnancy | 53 (27.3) | 158 (33.4) |  |
| Alcohol until pregnancy was known | 38 (19.6) | 103 (21.8) |  |
| Alcohol continued in pregnancy | 103 (53.1) | 212 (44.8) |  |
|  |  |  |  |
| **Child characteristics** |  |  |  |
| Gestational age at birth in weeks, median (95% range) | 40.4 (37.3 – 42.3) | 40.3 (36.0 – 42.3) | 0.710 |
| Premature birth, *n* (%) | 3 (1.3) | 14 (2.8) | 0.237 |
| Sex, *n* (%) |  |  | 0.040* |
| Male | 129 (57.3) | 249 (49.1) |  |
| Female | 96 (42.7) | 258 (50.9) |  |
| Birthweight, mean (±SD), gram | 3538.9 (453.5) | 3507.7 (515.0) | 0.434 |
| SGA, *n* (%) | 22 (9.8) | 62 (12.2) | 0.337 |
| LGA, *n* (%) | 22 (9.8) | 47 (9.3) | 0.828 |
| Low birthweight, *n* (%) | 2 (0.9) | 17 (3.4) | 0.053 |
| Macrosomia, *n* (%) | 31 (13.8) | 76 (15.0) | 0.668 |
| Values represent mean (SD), median (95% range) or number of participants (valid %). *p-value<0.05. ^a^ Dutch mothers with singleton live-born children with cord blood sampling for metabolomics without maternal urinary bisphenol measurement.  Premature birth, birth at gestational age <37 weeks; SGA, small for gestational age, gestational age adjusted birthweight <10^th^ percentile; LGA, large for gestational age, gestational age adjusted birthweight >90^th^ percentil; low birthweight, birthweight <2500 kilogram; macrosomia, birthweight >4000 kilogram. | | | |

**Supplementary table S9.** Association of maternal average and second trimester bisphenol A urinary concentrations in pregnancy with cord blood alkyl-lysophosphatidylcholines

|  | **Maternal average bisphenol A exposure** | | **Maternal second trimester bisphenol A exposure** | |
| --- | --- | --- | --- | --- |
| **Neonatal metabolites** | **Difference in neonatal metabolites (SDS) (95%CI)** | **P-value** | **Difference in neonatal metabolites (SDS) (95%CI)** | **P-value** |
| Lyso.PC.e C16:0 | 0.02 (-0.14, 0.17) | 0.851 | -0.03 (-0.20, 0.14) | 0.718 |
| Lyso.PC.e C18:0 | 0.00 (-0.16, 0.16) | 0.989 | 0.10 (-0.07, 0.27) | 0.232 |
| Lyso.PC.e C18:1 | 0.20 (0.04, 0.35) | 0.011* | 0.21 (0.05, 0.27) | 0.012* |
| Values represent regression coefficients (95% confidence interval) and corresponding p-values from linear regression models that reflect the difference in neonatal metabolite concentrations in SDS for an interquartile range increase in maternal bisphenol A concentration (in μmol/g creatinine). Model includes gestational age at intake, maternal age, parity, education, pre-pregnancy body mass index, smoking habits, alcohol consumption and maternal kcal intake. *p-value<0.05 | | | | |

**Supplementary table S10.** Association of maternal first trimester bisphenol A urinary concentrations in pregnancy with cord blood non-esterified fatty acids

|  | **Maternal first trimester bisphenol A** | |
| --- | --- | --- |
| **Neonatal metabolites** | **Differences in neonatal metabolites (SDS) (95%CI)** | **P-value** |
| NEFA C14:0 | -0.10 (-0.27, 0.06) | 0.214 |
| NEFA C14:1 | -0.07 (-0.23, 0.09) | 0.393 |
| NEFA C15:0 | -0.12 (-0.28, 0.05) | 0.161 |
| NEFA C16:0 | -0.18 (-0.34, -0.02) | 0.027* |
| NEFA C16:1 | -0.14 (-0.29, 0.02) | 0.090 |
| NEFA C16:2 | -0.16 (-0.32, -0.01) | 0.043* |
| NEFA C17:0 | -0.11 (-0.28, 0.05) | 0.171 |
| NEFA C17:1 | -0.11 (-0.28, 0.05) | 0.172 |
| NEFA C17:2 | -0.07 (-0.23, 0.10) | 0.414 |
| NEFA C18:0 | -0.11 (-0.27, 0.05) | 0.177 |
| NEFA C18:1 | -0.13 (-0.29, 0.03) | 0.101 |
| NEFA C18:2 | -0.16 (-0.32, 0.01) | 0.061 |
| NEFA C18:3 | -0.18 (-0.34, -0.01) | 0.034* |
| NEFA C19:1 | -0.10 (-0.26, 0.07) | 0.253 |
| NEFA C20:1 | -0.14 (-0.30, 0.02) | 0.094 |
| NEFA C20:2 | -0.16 (-0.33, 0.00) | 0.051 |
| NEFA C20:3 | -0.15 (-0.31, 0.01) | 0.073 |
| NEFA C20:4 | -0.11 (-0.28, 0.05) | 0.182 |
| NEFA C20:5 | -0.21 (-0.37, -0.04) | 0.013* |
| NEFA C22:3 | -0.18 (-0.34, -0.02) | 0.032* |
| NEFA C22:4 | -0.16 (-0.32, 0.00) | 0.054 |
| NEFA C22:5 | -0.14 (-0.30, 0.03) | 0.102 |
| NEFA C22:6 | -0.17 (-0.34, -0.01) | 0.036* |
| NEFA C24:0 | -0.16 (-0.32, 0.00) | 0.056 |
| NEFA C24:1 | -0.14 (-0.30, 0.03) | 0.104 |
| NEFA C24:2 | -0.17 (-0.33, -0.01) | 0.039* |
| NEFA C24:4 | -0.17 (-0.33, -0.01) | 0.038* |
| NEFA C24:5 | -0.20 (-0.36, -0.04) | 0.015* |
| NEFA C26:0 | -0.19 (-0.35, -0.03) | 0.024* |
| NEFA C26:1 | -0.16 (-0.32, 0.01) | 0.059 |
| NEFA C26:2 | -0.13 (-0.30, 0.03) | 0.109 |
| Values represent regression coefficients (95% confidence interval) and corresponding p-values from linear regression models that reflect the difference in neonatal metabolite concentrations in SDS for an interquartile range increase in maternal bisphenol A concentration (in μmol/g creatinine). Model includes gestational age at intake, maternal age, parity, education, pre-pregnancy body mass index, smoking habits, alcohol consumption and maternal kcal intake. *p-value<0.05 | | |

**Supplementary table S11.** Association of maternal average bisphenol S urinary concentrations in pregnancy with cord blood metabolic alkyl-lysophosphatidylcholines

|  | **Maternal average bisphenol S** | |
| --- | --- | --- |
| **Neonatal metabolites** | **Difference in neonatal metabolites (SDS) (95% CI)** | **P-value** |
| Lyso.PC.e C16:0 | -0.05 (-0.25, 0.14) | 0.616 |
| Lyso.PC.e C18:0 | -0.27 (-0.45, -0.08) | 0.006* |
| Lyso.PC.e C18:1 | -0.07 (-0.25, 0.12) | 0.496 |
| Values represent regression coefficients (95% confidence interval) and corresponding p-values from linear regression models that reflect the difference in neonatal metabolite concentrations in SDS for an interquartile range increase in maternal bisphenol S concentration (in μmol/g creatinine). Model includes gestational age at intake, maternal age, parity, education, pre-pregnancy body mass index, smoking habits, alcohol consumption and maternal kcal intake. *p-value<0.05 | | |

**Supplementary table S12.** Association of maternal first and third trimester bisphenol S urinary concentrations in pregnancy with cord blood acyl-carnitines

|  | **Maternal first trimester bisphenol S** | | **Maternal third trimester bisphenol S** | |
| --- | --- | --- | --- | --- |
| **Neonatal metabolites** | **Difference in neonatal metabolites (SDS) (95% CI)** | **P-value** | **Difference in neonatal metabolites (SDS) (95% CI)** | **P-value** |
| Carn.a C10:0 | 0.08 (-0.12, 0.27) | 0.443 | -0.06 (-0.17, 0.05) | 0.283 |
| Carn.a C10:1 | 0.21 (0.01, 0.40) | 0.035* | -0.08 (-0.19, 0.03) | 0.138 |
| Carn.a C12:0 | 0.18 (-0.01, 0.37) | 0.069 | -0.11 (-0.21, 0.00) | 0.051 |
| Carn.a C14:1 | 0.18 (-0.02, 0.37) | 0.077 | -0.11 (-0.22, 0.00) | 0.047* |
| Carn.a C14:2 | 0.27 (0.07, 0.46) | 0.007* | -0.15 (-0.26, -0.05) | 0.006* |
| Carn.a C15:0 | 0.24 (0.05, 0.43) | 0.016* | -0.12 (-0.23, -0.01) | 0.032* |
| Carn.a C16:0 | 0.20 (0.01, 0.40) | 0.044* | -0.08 (-0.19, 0.03) | 0.150 |
| Carn.a C16:0.Oxo | 0.31 (0.12, 0.51) | 0.002* | -0.03 (-0.14, 0.08) | 0.550 |
| Carn.a C16:1 | 0.22 (0.03, 0.42) | 0.025* | -0.14 (-0.25, -0.03) | 0.010* |
| Carn.a C16:2 | 0.16 (-0.04, 0.36) | 0.107 | -0.13 (-0.24, -0.03) | 0.017* |
| Carn.a C18:0 | 0.20 (0.00, 0.39) | 0.050* | -0.10 (-0.21, 0.01) | 0.082 |
| Carn.a C18:1 | 0.21 (0.01, 0.41) | 0.035* | -0.12 (-0.23, -0.02) | 0.026* |
| Carn.a C18:2 | 0.20 (0.00, 0.40) | 0.051 | -0.11 (-0.23, 0.00) | 0.044* |
| Carn.a C18:2.OH | 0.28 (0.08, 0.48) | 0.005* | -0.13 (-0.24, -0.02) | 0.024* |
| Carn.a C2:0 | 0.20 (0.01, 0.39) | 0.041* | -0.10 (-0.21, 0.00) | 0.056 |
| Carn.a C20:0 | 0.30 (0.10, 0.50) | 0.003* | -0.07 (-0.18, 0.05) | 0.254 |
| Carn.a C20:1 | 0.13 (-0.07, 0.32) | 0.219 | -0.11 (-0.22, 0.00) | 0.046* |
| Carn.a C20:3 | 0.37 (0.18, 0.56) | 0.000* | -0.12 (-0.23, -0.01) | 0.027* |
| Carn.a C20:4 | 0.35 (0.15, 0.54) | 0.000* | -0.10 (-0.21, 0.01) | 0.084 |
| Carn.a C3:0 | 0.06 (-0.14, 0.26) | 0.563 | -0.13 (-0.24, -0.02) | 0.025* |
| Carn.a C3:0.DC | 0.16 (-0.03, 0.36) | 0.104 | -0.05 (-0.16, 0.06) | 0.331 |
| Carn.a C4:0 | 0.16 (-0.03, 0.36) | 0.105 | -0.05 (-0.16, 0.06) | 0.394 |
| Carn.a C5:0 | 0.03 (-0.17, 0.23) | 0.770 | -0.08 (-0.19, 0.03) | 0.153 |
| Carn.a C6:0 | 0.14 (-0.06, 0.34) | 0.179 | -0.06 (-0.17, 0.05) | 0.293 |
| Carn.a C6:0.OH | 0.25 (0.06, 0.45) | 0.012* | -0.06 (-0.17, 0.05) | 0.266 |
| Carn.a C8:0 | 0.19 (-0.01, 0.38) | 0.058 | 0.02 (-0.09, 0.13) | 0.753 |
| Carn.a C8:1 | 0.11 (-0.09, 0.30) | 0.284 | -0.02 (-0.13, 0.09) | 0.696 |
| Carn.a C9:0 | 0.25 (0.05, 0.45) | 0.014* | -0.05 (-0.16, 0.06) | 0.363 |
| Values represent regression coefficients (95% confidence interval) and corresponding p-values from linear regression models that reflect the difference in neonatal metabolite concentrations in SDS for an interquartile range increase in maternal bisphenol S concentration (in μmol/g creatinine). Model includes gestational age at intake, maternal age, parity, education, pre-pregnancy body mass index, smoking habits, alcohol consumption and maternal kcal intake. *p-value<0.05. | | | | |

**Supplementary table S13.** Association of maternal third trimester bisphenol S urinary concentrations in pregnancy with cord blood non-esterified fatty acids

|  | **Maternal third trimester bisphenol S** | |
| --- | --- | --- |
| **Neonatal metabolites** | **Difference in neonatal metabolites (SDS) (95% CI)** | **P-value** |
| NEFA C14:0 | -0.10 (-0.21, 0.01) | 0.087 |
| NEFA C14:1 | -0.09 (-0.20, 0.03) | 0.128 |
| NEFA C15:0 | -0.07 (-0.18, 0.05) | 0.250 |
| NEFA C16:0 | -0.11 (-0.212, 0.00) | 0.056 |
| NEFA C16:1 | -0.08 (-0.18, 0.03) | 0.161 |
| NEFA C16:2 | -0.11 (-0.22, 0.00) | 0.051 |
| NEFA C17:0 | -0.05 (-0.16, 0.06) | 0.394 |
| NEFA C17:1 | -0.07 (-0.18, 0.04) | 0.203 |
| NEFA C17:2 | -0.08 (-0.20, 0.03) | 0.137 |
| NEFA C18:0 | -0.15 (-0.26, -0.04) | 0.006* |
| NEFA C18:1 | -0.09 (-0.20, 0.01) | 0.089 |
| NEFA C18:2 | -0.09 (-0.20, 0.02) | 0.123 |
| NEFA C18:3 | -0.11 (-0.22, 0.00) | 0.058 |
| NEFA C19:1 | -0.09 (-0.20, 0.02) | 0.106 |
| NEFA C20:1 | -0.15 (-0.26, -0.04) | 0.007* |
| NEFA C20:2 | -0.11 (-0.22, 0.00) | 0.050* |
| NEFA C20:3 | -0.09 (-0.20, 0.02) | 0.106 |
| NEFA C20:4 | -0.10 (-0.21, 0.02) | 0.094 |
| NEFA C20:5 | -0.10 (-0.21, 0.01) | 0.068 |
| NEFA C22:3 | -0.12 (-0.23, -0.02) | 0.026* |
| NEFA C22:4 | -0.07 (-0.18, 0.04) | 0.181 |
| NEFA C22:5 | -0.11 (-0.22, 0.00) | 0.056 |
| NEFA C22:6 | -0.13 (-0.24, -0.02) | 0.017* |
| NEFA C24:0 | 0.00 (-0.11, 0.11) | 0.956 |
| NEFA C24:1 | -0.09 (-0.20, 0.03) | 0.129 |
| NEFA C24:2 | -0.02 (-0.13, 0.10) | 0.788 |
| NEFA C24:4 | -0.04 (-0.15, 0.07) | 0.442 |
| NEFA C24:5 | -0.07 (-0.18, 0.04) | 0.205 |
| NEFA C26:0 | 0.07 (-0.04, 0.18) | 0.233 |
| NEFA C26:1 | 0.03 (-0.09, 0.14) | 0.645 |
| NEFA C26:2 | -0.01 (-0.12, 0.10) | 0.832 |
| Values represent regression coefficients (95% confidence interval) and corresponding p-values from linear regression models that reflect the difference in neonatal metabolite concentrations in SDS for an interquartile range increase in maternal bisphenol S concentration (in μmol/g creatinine). Model includes gestational age at intake, maternal age, parity, education, pre-pregnancy body mass index, smoking habits, alcohol consumption and maternal kcal intake. *p-value<0.05. | | |

**Supplementary table S14.** Association of maternal third trimester bisphenol F urinary concentrations in pregnancy with cord blood diacyl-phosphatidylcholines

|  | **Maternal third trimester bisphenol F** | |
| --- | --- | --- |
| **Neonatal metabolites** | **Difference in neonatal metabolites (SDS) (95% CI)** | **P-value** |
| PC.aa C30:0 | -0.11 (-0.28, 0.97) | 0.232 |
| PC.aa C30:3 | -0.26 (-0.44, -0.09) | 0.003* |
| PC.aa C32:0 | -0.21 (-0.38, -0.04) | 0.018* |
| PC.aa C32:1 | -0.15 (-0.33, 0.02) | 0.087 |
| PC.aa C32:2 | -0.17 (-0.34, 0.01) | 0.066 |
| PC.aa C32:3 | -0.11 (-0.29, 0.07) | 0.219 |
| PC.aa C34:1 | -0.22 (-0.40, -0.05) | 0.012* |
| PC.aa C34:2 | -0.13 (-0.30, 0.04) | 0.144 |
| PC.aa C34:3 | -0.18 (-0.35, -0.01) | 0.036* |
| PC.aa C34:4 | -0.16 (-0.34, 0.01) | 0.071 |
| PC.aa C34:5 | -0.29 (-0.46, -0.11) | 0.001* |
| PC.aa C36:0 | -0.15 (-0.32, 0.03) | 0.101 |
| PC.aa C36:1 | -0.29 (-0.46, -0.11) | 0.001* |
| PC.aa C36:2 | -0.24 (-0.41, -0.07) | 0.005* |
| PC.aa C36:3 | -0.18 (-0.36, -0.01) | 0.038* |
| PC.aa C36:4 | -0.18 (-0.36, -0.03) | 0.034* |
| PC.aa C36:5 | -0.12 (-0.29, 0.05) | 0.159 |
| PC.aa C36:6 | -0.05 (-0.23, 0.13) | 0.583 |
| PC.aa C38:0 | -0.11 (-0.28, 0.07) | 0.235 |
| PC.aa C38:2 | -0.04 (-0.22, 0.14) | 0.642 |
| PC.aa C38:3 | -0.20 (-0.38, -0.03) | 0.022* |
| PC.aa C38:4 | -0.16 (-0.33, 0.02) | 0.081 |
| PC.aa C38:5 | -0.21 (-0.38, -0.02) | 0.022* |
| PC.aa C38:6 | -0.11 (-0.28, 0.06) | 0.190 |
| PC.aa C40:0 | -0.01 (-0.18, 0.17) | 0.937 |
| PC.aa C40:1 | 0.04 (-0.14, 0.22) | 0.635 |
| PC.aa C40:2 | -0.12 (-0.28, 0.06) | 0.198 |
| PC.aa C40:3 | -0.20 (-0.37, -0.02) | 0.028* |
| PC.aa C40:4 | -0.14 (-0.31, 0.05) | 0.154 |
| PC.aa C40:5 | -0.17 (-0.35, 0.01) | 0.060 |
| PC.aa C40:6 | -0.13 (-0.30, 0.04) | 0.135 |
| PC.aa C42:0 | -0.08 (-0.25, 0.10) | 0.388 |
| PC.aa C42:5 | -0.05 (-0.22, 0.13) | 0.583 |
| PC.aa C43:6 | -0.03 (-0.21, 0.14) | 0.710 |
| PC.aa C44:12 | -0.15 (-0.33, 0.02) | 0.088 |
| Values represent regression coefficients (95% confidence interval) and corresponding p-values from linear regression models that reflect the difference in neonatal metabolite concentrations in SDS for an interquartile range increase in maternal bisphenol F concentration (in μmol/g creatinine). Model includes gestational age at intake, maternal age, parity, education, pre-pregnancy body mass index, smoking habits, alcohol consumption and maternal kcal intake. *p-value<0.05. | | |

**Supplementary table S15.** Association of maternal third trimester bisphenol F urinary concentrations in pregnancy with cord blood sphingomyelins

|  | **Maternal third trimester bisphenol F** | |
| --- | --- | --- |
| **Neonatal metabolites** | **Difference in neonatal metabolites (SDS) (95% CI)** | **P-value** |
| SM.a.C30.1 | -0.18 (-0.35, 0.00) | 0.048* |
| SM.a.C32.1 | -0.14 (-0.31, 0.03) | 0.117 |
| SM.a.C32.2 | -0.17 (-0.35, 0.00) | 0.047* |
| SM.a.C33.1 | -0.14 (-0.31, 0.03) | 0.106 |
| SM.a.C34.1 | -0.14 (-0.31, 0.03) | 0.113 |
| SM.a.C34.2 | -0.19 (-0.37, -0.02) | 0.028* |
| SM.a.C35.1 | -0.19 (-0.36, -0.02) | 0.032* |
| SM.a.C36.1 | -0.21 (-0.38, -0.03) | 0.020* |
| SM.a.C36.2 | -0.20 (-0.37, -0.03) | 0.024* |
| SM.a.C36.3 | -0.24 (-0.41, -0.06) | 0.008* |
| SM.a.C37.1 | -0.19 (-0.36, -0.01) | 0.037* |
| SM.a.C38.2 | -0.13 (-0.30, 0.04) | 0.134 |
| SM.a.C38.3 | -0.12 (-0.30, 0.05) | 0.165 |
| SM.a.C39.1 | -0.21 (-0.38, -0.04) | 0.016* |
| SM.a.C39.2 | -0.08 (-0.26, 0.10) | 0.370 |
| SM.a.C40.2 | -0.13 (-0.30, 0.04) | 0.142 |
| SM.a.C40.5 | -0.14 (-0.31, 0.03) | 0.098 |
| SM.a.C41.1 | -0.13 (-0.30, 0.04) | 0.129 |
| SM.a.C41.2 | -0.14 (-0.31, 0.04) | 0.122 |
| SM.a.C42.1 | -0.17 (-0.35, 0.00) | 0.054 |
| SM.a.C42.2 | -0.21 (-0.38, -0.03) | 0.019* |
| SM.a.C42.3 | -0.14 (-0.31, 0.03) | 0.113 |
| SM.a.C42.4 | -0.25 (-0.42, -0.08) | 0.005* |
| SM.a.C42.6 | -0.18 (-0.35, -0.01) | 0.043* |
| SM.a.C43.1 | -0.10 (-0.27, 0.08) | 0.279 |
| SM.a.C43.2 | -0.18 (-0.35, -0.01) | 0.038* |
| SM.a.C44.6 | -0.03 (-0.21, 0.15) | 0.734 |
| SM.e.C36.2 | -0.06 (-0.23, 0.12) | 0.522 |
| SM.e.C38.3 | -0.15 (-0.33, 0.03) | 0.100 |
| SM.e.C40.5 | -0.03 (-0.20, 0.15) | 0.764 |
| Values represent regression coefficients (95% confidence interval) and corresponding p-values from linear regression models that reflect the difference in neonatal metabolite concentrations in SDS for an interquartile range increase in maternal bisphenol F concentration (in μmol/g creatinine). Model includes gestational age at intake, maternal age, parity, education, pre-pregnancy body mass index, smoking habits, alcohol consumption and maternal kcal intake. *p-value<0.05. | | |

**Supplementary figure S1.** Direct Acyclic Graph analysis on the hypothesized relationship between maternal bisphenol exposure, neonatal metabolites and the covariates.
